# Catalytic Nitric Oxide‐Generating Selenium Nanoparticle‐Loaded Hydrogels With Anti‐Inflammatory and Pro‐Wound‐Healing Properties

**DOI:** 10.1002/smsc.70409

**Published:** 2026-09-21

**Authors:** Shu Geng, Kang Lin, Qingqing Fan, Yulong Sun, Federico Mazur, Rona Chandrawati

**Affiliations:** ^1^ School of Chemical Engineering and Australian Centre for Nanomedicine (ACN) The University of New South Wales (UNSW Sydney) Sydney NSW Australia

**Keywords:** anti‐inflammatory, catalysis, hydrogel, nitric oxide, selenium nanoparticles

## Abstract

Nitric oxide’s (NO) crucial role in various physiological processes, such as vascular regulation, inflammation, and wound healing, has led to the development of various NO delivery platforms. However, delivering NO in a controlled and sustained manner is challenging due to its short half‐life of less than 5 s. To address this challenge, we synthesized polyacrylamide (PAAm) hydrogels loaded with selenium nanoparticles (SeNPs) or polydopamine‐coated selenium nanoparticles (Se@PDA NPs), which can catalytically generate NO from both endogenous and exogenous NO donors. Structural characterization confirmed that nanoparticle loading did not alter the intrinsic properties of the PAAm‐gel. By adjusting the concentration of NPs loaded within the hydrogels, tunable NO generation was achieved. Both Se‐gel and Se@PDA‐gel exhibited sustained NO generation, with Se@PDA‐gel demonstrating superior recyclability and stability, even after long‐term storage under various conditions. The hydrogels exhibited excellent biocompatibility, and their biological activity was validated by elevated interleukin‐10 (IL‐10) and α‐smooth muscle actin (α‐SMA) expression, indicating anti‐inflammatory effects and potential for wound healing, respectively. These findings establish Se@PDA‐gel as a promising platform for controlled and sustained NO delivery, offering therapeutic potential in wound healing and anti‐inflammatory therapies, particularly for cardiovascular and infectious diseases.

## Graphical abstract

Polydopamine‐coated selenium nanoparticle (Se@PDA)‐loaded hydrogels enable sustained and tunable catalytic nitric oxide (NO) generation from *S*‐nitrosoglutathione (GSNO). The hydrogels exhibit excellent stability, recyclability, and biocompatibility, while elevating IL‐10 expression in endothelial cells and smooth muscle cells and increasing α‐SMA expression in fibroblasts, demonstrating anti‐inflammatory and pro‐wound‐healing properties.

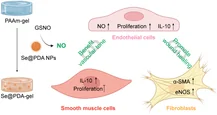

## 1 Introduction

Nitric oxide (NO) is an important gaseous signaling molecule involved in various physiological processes. Its significance was first recognized in the cardiovascular system, where it acts as a vasodilator, relaxing vascular smooth muscle and lowering blood pressure [1]. Additionally, NO has antithrombotic properties [2], inhibiting platelet aggregation [3] and reducing the risk of blood clots, heart attacks, and strokes. Its anti‐inflammatory [4] and anti‐proliferative [5] properties also help prevent atherosclerosis by inhibiting the buildup of plaques in the arteries and suppressing excessive proliferation of smooth muscle cells. Beyond the cardiovascular system, NO plays important roles in neurotransmission [6] and immune responses [7]. In conditions such as multiple sclerosis, a chronic autoimmune disorder of the central nervous system, NO is often associated with neurotoxicity. Elevated levels of NO, produced by activated immune cells like macrophages and microglia during inflammatory responses, can cause direct neuronal damage, contributing to the neurodegenerative progression of multiple sclerosis. In wound healing, NO promotes reepithelialization [8], enhances the migration and proliferation of fibroblasts [9], and supports collagen production [10] and deposition [11]. In the cancerous environment, depending on the concentration, NO can have tumor‐promoting or tumor‐suppressing effects [12]. Low levels of NO may support tumor growth by promoting angiogenesis, while high concentrations can lead to the formation of reactive nitrogen species, inducing oxidative stress and contributing to cytostatic or cytotoxic effects. Despite NO’s broad physiological significance, its clinical application is challenging due to its highly reactive nature and short half‐life (<5 s) [13].

Various macromolecular NO donors have been developed to stabilize and deliver NO effectively. These include *S*‐nitrosothiols (RSNO) and diazeniumdiolates (NONOates), which can be encapsulated [14] within or modified [15] onto NO delivery vehicles. These donors release NO in response to various triggers, such as light [16], pH changes [17], oxidants, reductants, and thiols [18], allowing for controlled and targeted NO delivery. This approach has been applied in therapeutic applications, including antimicrobial [19], anticancer [20], and wound healing [21]. However, donor‐based systems are inherently limited by finite NO payloads. Once depleted, NO release ceases, making them unsuitable for applications requiring long‐term or continuous therapeutic NO levels, such as chronic vascular implants, persistent infections, or impaired wound healing. To address this limitation, catalytic approaches have emerged, using catalysts to decompose endogenous NO donors, thereby achieving in situ sustained NO generation. Copper ions were the first to be reported for their capacity to decompose the endogenous NO donor *S*‐nitrosoglutathione (GSNO) to generate NO [22]. Following this, copper‐based nanomaterials and coatings have been developed, demonstrating biofilm dispersal [23] and antithrombotic [24] properties. In addition to metal materials, selenium compounds have also demonstrated the capacity for NO generation [25]. We have previously demonstrated that selenium nanoparticles (SeNPs) exhibit high NO‐generating ability with low cytotoxicity [26]. Their catalytic performance was evaluated through kinetic studies and recycling tests, demonstrating concentration‐dependent catalytic activity, enhanced NO generation in the presence of glutathione (GSH), and sustained catalytic performance over multiple reaction cycles. Moreover, by coating these nanoparticles with polydopamine, core/shell nanoparticles (Se@PDA NPs) offer improved catalyst stability in physiological conditions and enhanced cellular uptake, facilitating efficient intracellular NO generation [27]. However, despite the demonstrated catalytic activity of SeNPs and Se@PDA NPs, there remain major translational challenges: (1) the catalyst must be localized at the treatment site, (2) the catalytic activity must be sustained and controllable, and (3) the platform must be mechanically and biologically compatible with the target tissue.

Hydrogels provide an attractive solution to these challenges. Their mechanical properties can be precisely tuned, allowing them to mimic diverse tissues, and their high biocompatibility and ability to encapsulate functional nanomaterials make them excellent candidates for creating localized reservoirs of catalytic NO activity. Polyacrylamide (PAAm) hydrogels, in particular, offer broad versatility and can be tailored for specific applications by adjusting the composition of the reaction mixture. For example, the mechanical properties of PAAm hydrogels can be fine‐tuned, with Young’s modulus ranging from 0.2 to 145 kPa, depending on the concentrations of acrylamide (AAm) and the cross‐linker bisacrylamide (Bis‐AAm) [28, 29]. This wide range of stiffness of hydrogels enhances their applicability in tissue engineering by enabling them to match the mechanical properties of target tissues. For instance, softer hydrogels can mimic brain [30] and lung tissue [31], while stiffer hydrogels are better suited for mimicking muscle [32] and bone tissue [33]. Additionally, their adaptable physicochemical properties support applications in tissue engineering, stem cell differentiation [34], and smart drug delivery systems [35]. The high biocompatibility of PAAm gels further enhances their suitability for a wide range of biomedical applications [36].

Herein, we synthesized a catalytic PAAm hydrogel loaded with SeNPs or Se@PDA core/shell NPs to facilitate NO generation from both endogenous (GSNO) and exogenous (*S*‐Nitroso‐*N*‐Acetyl‐DL‐Penicillamine, SNAP) NO donors. The ability to tune NO release was achieved by varying the concentrations of SeNPs or Se@PDA NPs within the hydrogel matrix. We also assessed the system’s sustained NO generation, stability, and biocompatibility. Cellular effects were investigated by measuring intracellular NO levels, the expression of the anti‐inflammatory cytokine interleukin‐10 (IL‐10) in human umbilical vein endothelial cells (HUVECs) and human coronary artery smooth muscle cells (HCASMCs), and the expression of α‐smooth muscle actin (α‐SMA) in NIH 3T3 fibroblasts. These findings highlight the potential of NO‐generating hydrogels in treating cardiovascular diseases and promoting wound healing. In this study, we developed a stable, biocompatible, and therapeutically versatile NO‐generating hydrogel, showcasing its relevance in NO‐mediated anti‐inflammatory and tissue repair processes.

## 2 Results and Discussion

### 2.1 Characterization of NPs and Hydrogels

Hydrogels are widely used in biomedical applications owing to their tunable mechanical properties and high biocompatibility. Incorporating NPs into hydrogels can impart additional properties while minimizing direct NP‐tissue interactions, thereby mitigating potential cytotoxic effects. In this study, hydrogels were synthesized using free‐radical polymerization, with AAm as the monomer, bis‐AAm as the cross‐linker, TEMED as the catalyst, and APS as the initiator. Three different hydrogels were prepared: i) PAAm‐gel, used as the control group, ii) Se‐gel, hydrogels loaded with SeNPs, and iii) Se@PDA‐gel, hydrogels loaded with Se@PDA NPs. The morphological and elemental characteristics of SeNPs and Se@PDA NPs were shown in Figure 1a,b, respectively. Compared with bare SeNPs, Se@PDA NPs exhibit a slightly increased particle size and rougher surface. High‐angle annular dark‐field scanning transmission electron microscopy (HAADF–STEM) elemental mapping demonstrates a homogeneous Se distribution in both samples, with additional carbon, nitrogen, and oxygen signals observed in Se@PDA NPs, confirming successful PDA coating. After the hydrogels were formed, they were cut and freeze‐dried in liquid nitrogen, and their cross‐sections were characterized using scanning electron microscopy (SEM). All hydrogels exhibited a porous internal structure, and SeNP or Se@PDA NP incorporation did not alter pore morphology (Figure 1c). SEM‐EDS confirmed the successful loading and dispersion of SeNPs or Se@PDA NPs. The energy‐dispersive spectroscopy (EDS) spectra (Figure 1d,e) displayed distinctive peaks of Se at 1.4 keV, and EDS mapping showed that the NPs were evenly distributed. Next, Raman spectroscopy was used to analyze the chemical structure of the hydrogels (Figure 1f). The peak assignments of the PAAm‐gel are listed in Table S1 and correlate well with the literature [37, 38, 39]. No new shifts were observed in Se‐gel and Se@PDA‐gel, indicating that SeNP and Se@PDA NP incorporation did not impact the chemical structure of the PAAm hydrogel. This suggests that the NPs were physically adsorbed within the hydrogels without altering their chemical composition. Fourier‐transform infrared spectroscopy (FT‐IR) was used as a complementary technique to further characterize the structure of the hydrogels (Figure 1g and Table S2). All hydrogels exhibited a broad peak at 3320–3340 cm^−1^, a sharp peak at 1640–1650 cm^−1^, and 1400–1420 cm^−1^, corresponding to N─H, C═O, and C─H stretching, respectively. Moreover, X‐ray diffraction (XRD) analysis was used to examine the crystalline structure of the hydrogels (Figure 1h). All three types of hydrogels exhibited a broad peak at 20°–30°, indicating that the hydrogels were amorphous in structure. Finally, X‐ray photoelectron spectroscopy (XPS) was carried out to characterize the surface properties of the hydrogels by examining their elemental composition and chemical states. The survey scans (Figure 1i) indicated the presence of oxygen, nitrogen, and carbon elements. Due to the low concentration of NPs loaded and their encapsulation within the hydrogels, selenium was not detected on the surface of the hydrogels. Table S3 summarizes the positions and assignments of all the XPS peaks. The high‐resolution C1s spectra (Figure S1a) showed three peaks at around 284.8, 286.1, and 287.9 eV, which were attributed to C/C─H, C─O, and N─C═O, respectively. The N1s spectra (Figure S1b) of Se‐gel and PAAm‐gel showed a single peak at 399.5 eV, which was assigned to the amide group, whereas the N1s spectra of Se@PDA‐gel had two other minor peaks at 400.6 and 402.1 eV, which were attributed to the polydopamine coating on the SeNP surface. The O1s spectra (Figure S1c) exhibited two peaks at approximately 532.2 and 532.8 eV, corresponding to C═O and C─O bonds, respectively. Collectively, the XPS results further confirmed that NP loading did not introduce new chemical bonds within the PAAm network.

**FIGURE 1:**
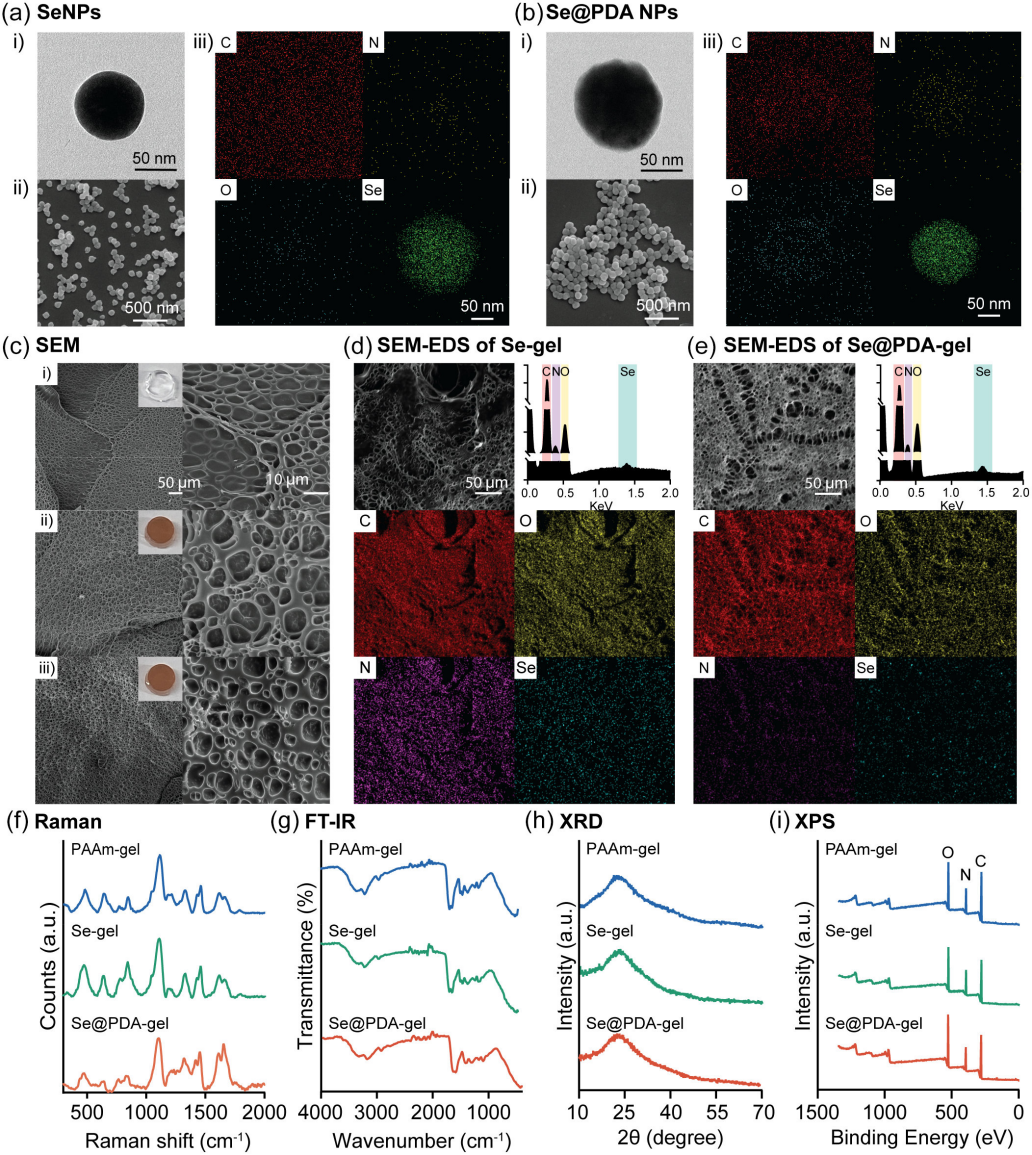
Characterization of (a) SeNPs and (b) Se@PDA NPs, including (i) TEM, (ii) SEM, and (iii) HAADF–STEM elemental mapping of C, N, O, and Se. (c) SEM images of (i) PAAm‐gel, (ii) Se‐gel, and (iii) Se@PDA‐gel with corresponding optical images inset. SEM‐EDS analysis of (d) Se‐gel and (e) Se@PDA‐gel, including EDS spectrum and elemental mapping of C, N, O, and Se. (f) Raman, (g) FT‐IR, (h) XRD, and (i) XPS analysis of PAAm‐gel, Se_0.15_‐gel, and Se@PDA_0.15_‐gel.

### 2.2 Mechanical Properties of Hydrogels

We next investigated the mechanical properties of PAAm‐gel, Se‐gel, and Se@PDA‐gel to determine whether incorporating nanoparticles would affect the behavior of the hydrogel matrix. All three hydrogels exhibited water contents exceeding 80% (Figure 2a), closely resembling biological tissues. The high water content provides an aqueous environment for cells and sensitive drugs (therapeutic proteins and growth factors) and prevents them from denaturation, enhancing their applications in biomedical fields [40]. Additionally, the swelling ratio (SR), a crucial factor in determining the stability of hydrogels, was assessed at 37 °C, as it can significantly impact the mechanical properties [41], drug release kinetics [42], degradability [43], and biocompatibility [44]. For instance, high‐swelling hydrogels (SR > 150%) exhibit high wound exudate absorption capacity, enhanced cell recruitment and migration, and superior drug entrapment, diffusion, and release capacities [45]. However, in biomedical applications such as scaffolds for organ regeneration in tissue engineering, hydrogels with a high SR might compress or even damage surrounding tissues [46]. Nonswelling hydrogels (SR < 150%) offer excellent dimensional stability and sustained mechanical strength, but they typically have lower cell attachment and reduced drug loading [45]. Therefore, the SR of hydrogels must be carefully tailored to the specific requirements of the intended application. As depicted in Figure 2b, the SR of hydrogels reached approximately 10% for PAAm‐gel and Se‐gel and 20% for Se@PDA‐gel, with no further water absorption observed over the subsequent 4 days. The SR of the freeze‐dried hydrogels, denoted as SR_d_, ranged from 600% to 800%, remaining stable over the subsequent 4 days. The SR was also measured at room temperature, a crucial parameter for ex vivo applications, such as wound dressings. The SR at room temperature followed a similar trend to that observed at body temperature (Figure S2), demonstrating their stable swelling behaviors across both ambient and physiological temperatures. Furthermore, compression tests (Figure 2c) were conducted to evaluate the mechanical properties of the hydrogels. They exhibited high toughness, breaking only when deformation approached 75% under stress exceeding 0.25 MPa (Figure S3). Overall, the incorporation of NPs into the PAAm gel had a negligible effect on their mechanical properties.

**FIGURE 2:**
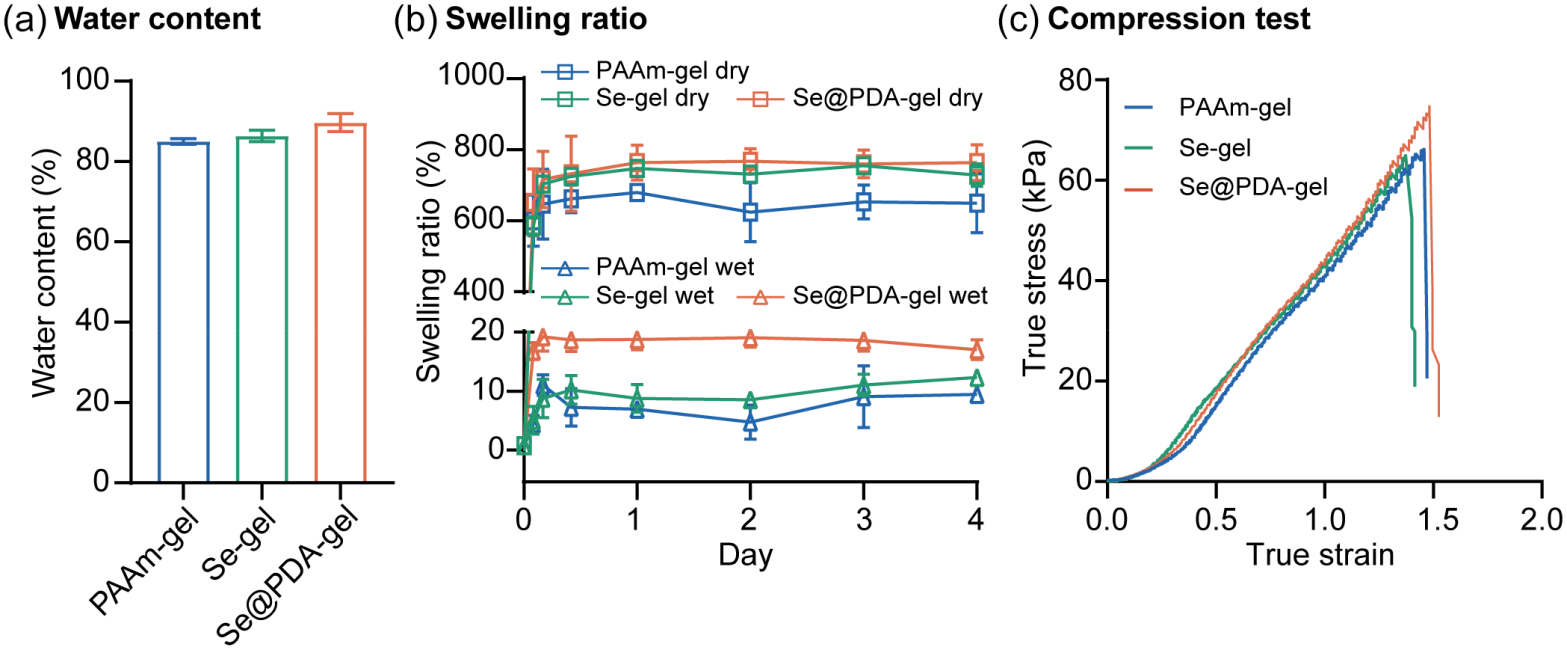
(a) Water content, (b) swelling ratio at 37 °C, and (c) compression test of PAAm‐gel, Se‐gel, and Se@PDA‐gel. *n* = 3; error bars represent standard deviation.

### 2.3 Tunable NO Generation

SeNPs and Se@PDA NPs have been demonstrated as effective catalysts for NO generation from endogenous and exogenous RSNOs [26, 27]. As illustrated in Figure S4a, GSNO adsorbs onto the SeNP surface, where strong affinity between Se and S facilitates S─N bond cleavage, leading to NO release and formation of glutathione thiyl radicals (GS•), which subsequently recombine to form glutathione disulfide (GSSG). In the presence of GSH, the mechanism is expected to resemble that of organoselenium systems (Figure S4b) [47], in which GSH reduces the Se surface to active Se─H/Se^−^ species, thereby sustaining catalytic NO generation. Concurrently, GSH also promotes S─N bond cleavage of RSNOs via transnitrosation [48], further accelerating NO generation. To evaluate whether this catalytic behavior is retained after incorporation into a hydrogel matrix, the NO‐generating capacities of Se‐gel and Se@PDA‐gel were investigated using an amperometric sensor equipped with a NO‐sensitive probe. To mimic a biological environment, the endogenous NO donor GSNO (10 µM) and GSH (1 mM) at bio‐relevant concentrations were added to PBS (pH 7.4) buffer containing one piece of hydrogel sample. NO generation was monitored in real time over a 40 min period. As illustrated in Figure 3a, both Se_0.15_‐gel and Se@PDA_0.15_‐gel produced markedly higher NO levels compared to the control. NO is a pleiotropic regulator capable of exerting opposite effects under various conditions. Therefore, achieving tunable NO generation is of great significance, enabling its potential across various therapeutic applications. To explore this, hydrogels loaded with different concentrations of SeNPs or Se@PDA NPs were incubated with GSNO (50 µM) and GSH (1 mM) in PBS. After 15, 30, and 60 min, a Griess assay was carried out to determine the cumulative NO levels (Figure S5a). The NO generation kinetics were not significantly affected by using Se‐gel with SeNPs loaded at various concentrations. However, in the case of Se@PDA‐gel, the kinetics can be modulated by adjusting the NP concentration. Since the NO generation reached saturation after 30 min of incubation, we compared the 15 min cumulative NO generation, which showed no significant differences among the Se‐gel groups (Figure 3bi) but exhibited an increasing trend for the Se@PDA‐gel groups (Figure 3bii). Additionally, the ability to generate NO from an exogenous NO donor was explored using SNAP, an *S*‐nitrosothiol structurally similar to GSNO. As illustrated in Figure 3c, the peak NO levels exceeded 2.5 µM upon the introduction of Se‐gel or Se@PDA‐gel, whereas the control remained below 0.5 µM. Similarly, tunable NO generation was explored by analyzing time‐dependent cumulative NO generation (Figure S5b). Both Se‐gel and Se@PDA‐gel exhibited a concentration‐dependent trend, with higher levels of NO generated using hydrogels loaded with higher concentrations of NPs (Figure 3d). Although Se‐gel loaded with 0.05–0.3 mg mL^−1^ SeNPs did not show statistically significant differences (Figure 3di), Se@PDA‐gel displayed robust, progressive increases across the entire 0.01–0.3 mg mL^−1^ range (Figure 3dii). Overall, both Se‐gel and Se@PDA‐gel demonstrated the capacity to generate NO from endogenous and exogenous NO donors, with Se@PDA‐gel offering pronounced and controllable concentration‐dependent behavior. Moreover, the NO‐generating activity of Se‐gel and Se@PDA‐gel was compared with that of free SeNPs and Se@PDA NPs (Figure S6). Se@PDA NPs exhibited lower NO‐generating activity than bare SeNPs at the same concentration, likely due to partial shielding of catalytically active Se sites by the PDA coating. However, upon incorporation into the hydrogel, the activity of SeNPs decreased significantly, whereas Se@PDA NPs maintained comparable performance. This suggests that bare SeNPs are more susceptible to mass transfer limitations within the hydrogel matrix, whereas the PDA coating enhances interfacial compatibility and substrate accessibility. This improvement is attributed to the increased hydrophilicity of PDA [49], which promotes more uniform nanoparticle dispersion and enhances local water retention within the hydrogel, thereby facilitating the diffusion of GSNO and GSH to the catalytic interface and ultimately mitigating diffusion constraints to preserve catalytic activity.

**FIGURE 3:**
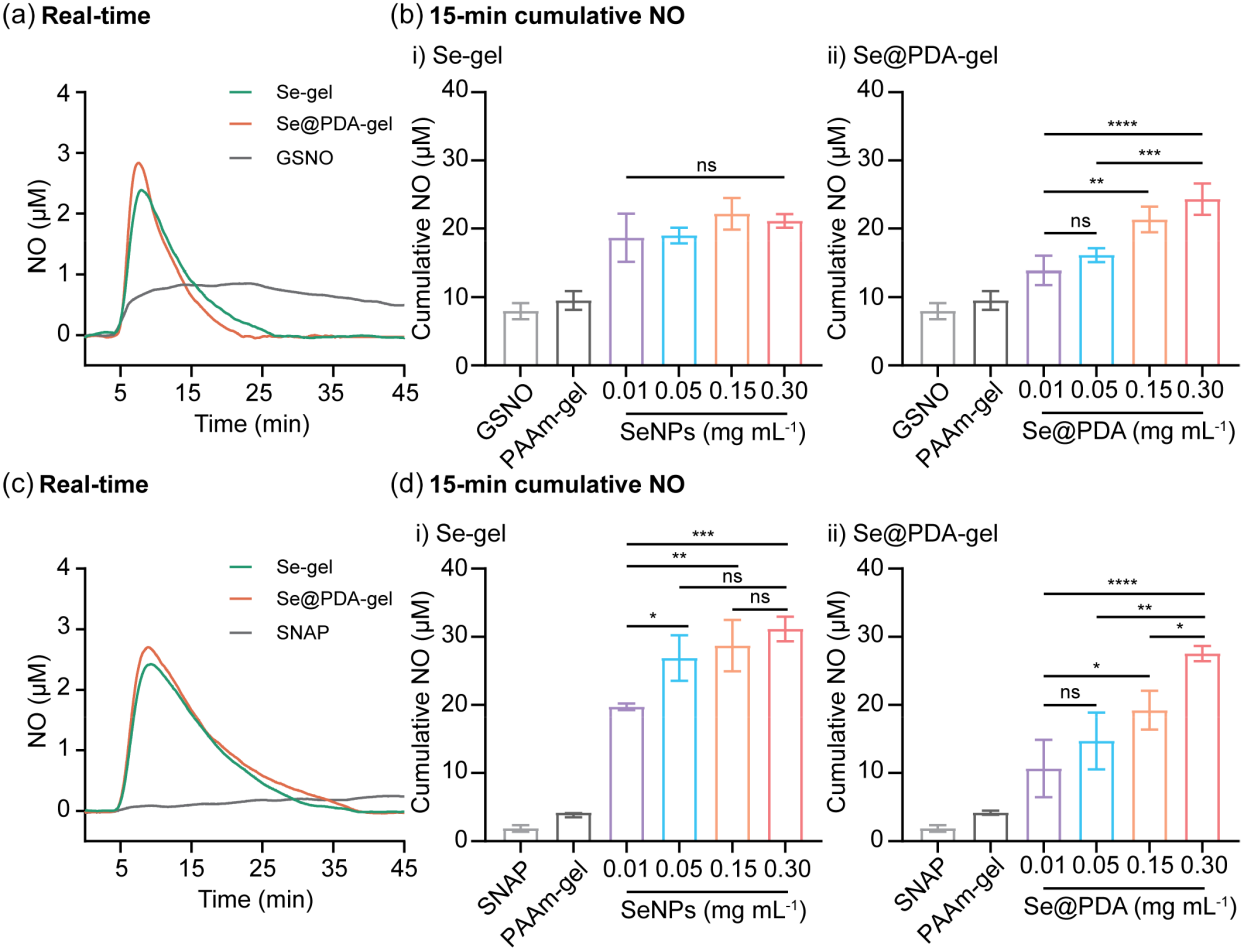
Catalytic NO generation using Se‐gel or Se@PDA‐gel. Real‐time NO generation catalyzed by Se_0.15_‐gel or Se@PDA_0.15_‐gel from (a) GSNO or (c) SNAP (10 µM), in the presence of GSH (1 mM). 15 min cumulative NO generation from (b) GSNO or (d) SNAP (50 µM) catalyzed by (i) Se‐gel or (ii) Se@PDA‐gel, with NPs loaded at various concentrations (0, 0.01, 0.05, 0.15, or 0.3 mg mL^−1^) in the presence of GSH (1 mM). Statistical significance was calculated using one‐way ANOVA, ns = no significance, *p < 0.05, **p < 0.01, ***p < 0.001, ****p < 0.0001. *n* = 3; error bars represent standard deviation.

### 2.4 Sustained NO Generation and Stability

The recyclability and stability of biomaterials are critical factors that affect their practical use, especially if applied in implantable systems. To evaluate these aspects, we first investigated the recyclability of the hydrogel using a NO‐sensitive electrochemical probe by measuring its real‐time NO generation over five successive doses of GSNO (10 µM) in the presence of GSH (1 mM) [50]. As shown in Figure 4a, Se‐gel and Se@PDA‐gel maintained their catalytic efficiency over five consecutive reactions, with a slight increase in [NO]_peak_ that may be attributed to the accumulation of GSH over time. Similarly, both hydrogels sustained real‐time NO generation from SNAP over five consecutive reaction cycles (Figure 4b). The recyclability of the hydrogels was further examined by measuring the time‐dependent cumulative NO generation over five consecutive days. After each cycle, the hydrogels were washed with PBS and stored in PBS overnight at 4 °C. The catalytic activities during the first cycle are presented in Figure S5, while the subsequent cycles of NO generation from GSNO are summarized in Figure S7. NO generation catalyzed by Se‐gel or Se@PDA‐gel reached saturation within 30 min, with significant differences observed after 15 min incubation. Specifically, the NO‐generating capacity of Se_0.15_‐gel from GSNO showed a significant decrease by the fifth cycle (Figure 4ci). In contrast, Se@PDA_0.15_‐gel demonstrated greater stability, with no significant reduction in NO generation ability after five reaction cycles (Figure 4cii). Given that GSNO is more reactive than SNAP under these conditions, as indicated by the higher control values, NO generation from GSNO catalyzed by Se_0.15_‐‍gel and Se@PDA_0.15_‐gel did not show considerable differences. Consequently, cumulative NO generation from SNAP was also examined (Figure S8). As recycling increased, the catalytic activity of Se_0.15_‐gel decreased (Figure 4di). However, Se@PDA_0.15_‐gel maintained a high NO‐generating ability, with no significant difference compared to the first cycle (Figure 4dii). The recyclability of Se‐gel and Se@PDA‐gel with NPs loaded at other concentrations is summarized in Figure S9, presenting the same trend as observed with Se_0.15_‐gel and Se@PDA_0.15_‐gel. Our previous findings demonstrated that Se@PDA NPs remained stable after five reaction cycles, as evidenced by their well‐dispersed morphology and intact PDA shell observed in SEM and TEM images, whereas bare SeNPs showed aggregation after recycling [27]. Incorporation of SeNPs into the hydrogel matrix did not adequately preserve their structural integrity or catalytic activity, while Se@PDA maintained both stability and functionality after hydrogel incorporation. Further characterization was conducted to analyze the structure of the hydrogels after incubation. As illustrated in Figure S10, the broad XRD peak at 20°–30° indicates that the PAAm‐gel exhibited an amorphous structure consistent with the literature [51]. The Se@PDA‐gel retained its amorphous structure, whereas the Se‐gel shifted to a crystalline structure, showing sharp peaks corresponding to the Se pattern. This structural shift correlates with the decreased catalytic activity of Se‐gel, consistent with our previous findings that amorphous SeNPs exhibit higher NO‐generating capacity compared to their crystalline counterparts [26]. Moreover, ICP analysis revealed minimal selenium leaching after incubation in PBS for up to 7 days, with only 0.149% released from Se‐gel and 0.128% from Se@PDA‐gel (Table S4). This minimal leaching, combined with physical encapsulation of NPs in the PAAm‐gel, reduces direct contact of NPs with tissues and helps mitigate potential cytotoxic effects.

**FIGURE 4:**
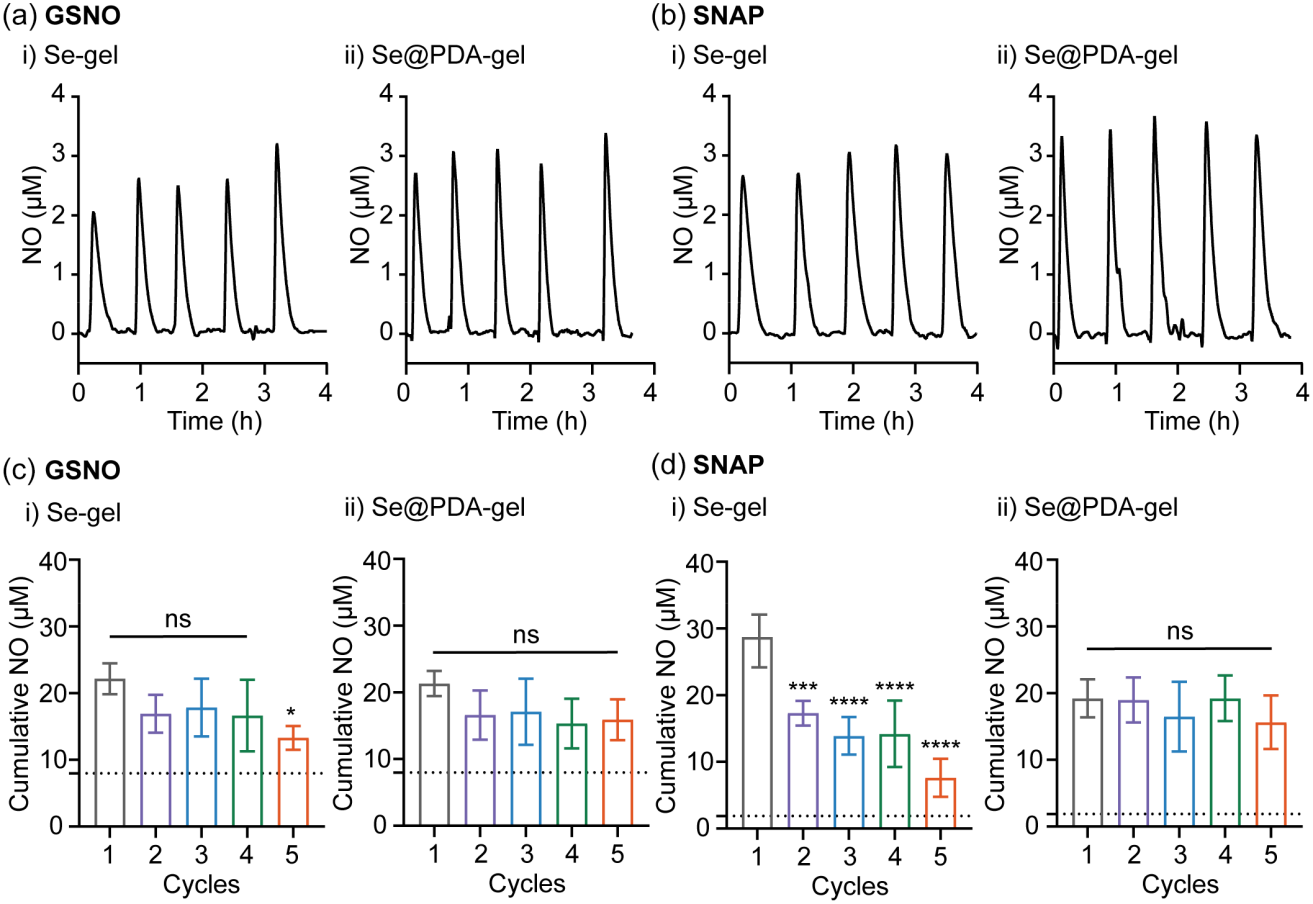
Sustained NO generation from (a) GSNO or (b) SNAP (10 µM) by (i) Se_0.15_‐gel or (ii) Se@PDA_0.15_‐gel in the presence of GSH (1 mM). 15 min cumulative NO generation catalyzed from (c) GSNO or (d) SNAP (50 µM) by (i) Se_0.15_‐gel or (ii) Se@PDA_0.15_‐gel in the presence of GSH (1 mM) over five reaction cycles. The dashed line represents the control (GSNO or SNAP only) in the corresponding conditions. Statistical significance was calculated using two‐way ANOVA, ns = no significance, *p < 0.05, **p < 0.01, ***p < 0.001, ****p < 0.0001. *n* = 3; error bars represent standard deviation.

In addition to recyclability, long‐term stability is another important factor to consider when evaluating applicability. As such, Se@PDA‐gels were stored for 2 months under two conditions: (i) at 4 °C in PBS and (ii) at room temperature after being freeze‐dried. Then, a Griess assay was conducted to measure the NO‐generating capacity of Se@PDA‐gel from both GSNO (Figure 5a) and SNAP (Figure 5b). Only Se@PDA‐gel was tested due to its superior performance in recycling tests. No significant difference in NO generation was observed across all conditions, indicating high stability in both wet and dry states. Se@PDA‐gel demonstrated sustained NO generation and long‐term stability; therefore, the cytotoxicity and cellular effects of Se@PDA‐gel with NPs loaded at various concentrations, with Se_0.3_‐gel included as a comparative control, will be explored in the following sections to evaluate their potential for biomedical applications.

**FIGURE 5:**
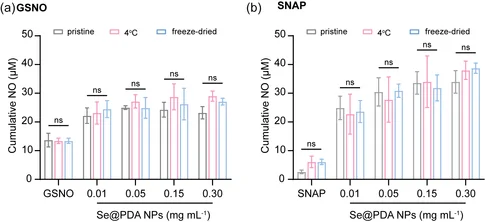
Cumulative NO generation from (a) GSNO or (b) SNAP (50 µM) catalyzed by pristine Se@PDA‐gel, Se@PDA‐gel stored at 4 °C for 2 months, or freeze‐dried Se@PDA‐gel stored at room temperature for 2 months, with NPs loaded at various concentrations (0, 0.01, 0.05, 0.15, or 0.3 mg mL^−1^). Statistical significance was calculated using two‐way ANOVA, ns = no significance. *n* = 3; error bars represent standard deviation.

### 2.5 Cytotoxicity

NO has been recognized as an important gaseous signaling molecule in the cardiovascular system due to its role in smooth muscle cell‐mediated vasodilation. Therefore, HCASMCs were selected as a model to assess the biocompatibility of these hydrogels. PAAm‐gel, Se‐gel, and Se@PDA‐gel loaded with NPs at concentrations ranging from 0.01 to 0.3 mg mL^−1^ were placed into 3D‐printed cell culture plate inserts (Figure S11) and incubated in the medium with cells. After 48 h of incubation, the Live/Dead assay was employed to measure cell viability, with a negligible number of dead cells detected, demonstrating the high biocompatibility of these hydrogels (Figure 6a). Quantitative analysis of the Live/Dead assay revealed that the average cell viability exceeded 94%, with no significant differences observed between the control and hydrogel‐treated groups (Figure 6b).

**FIGURE 6:**
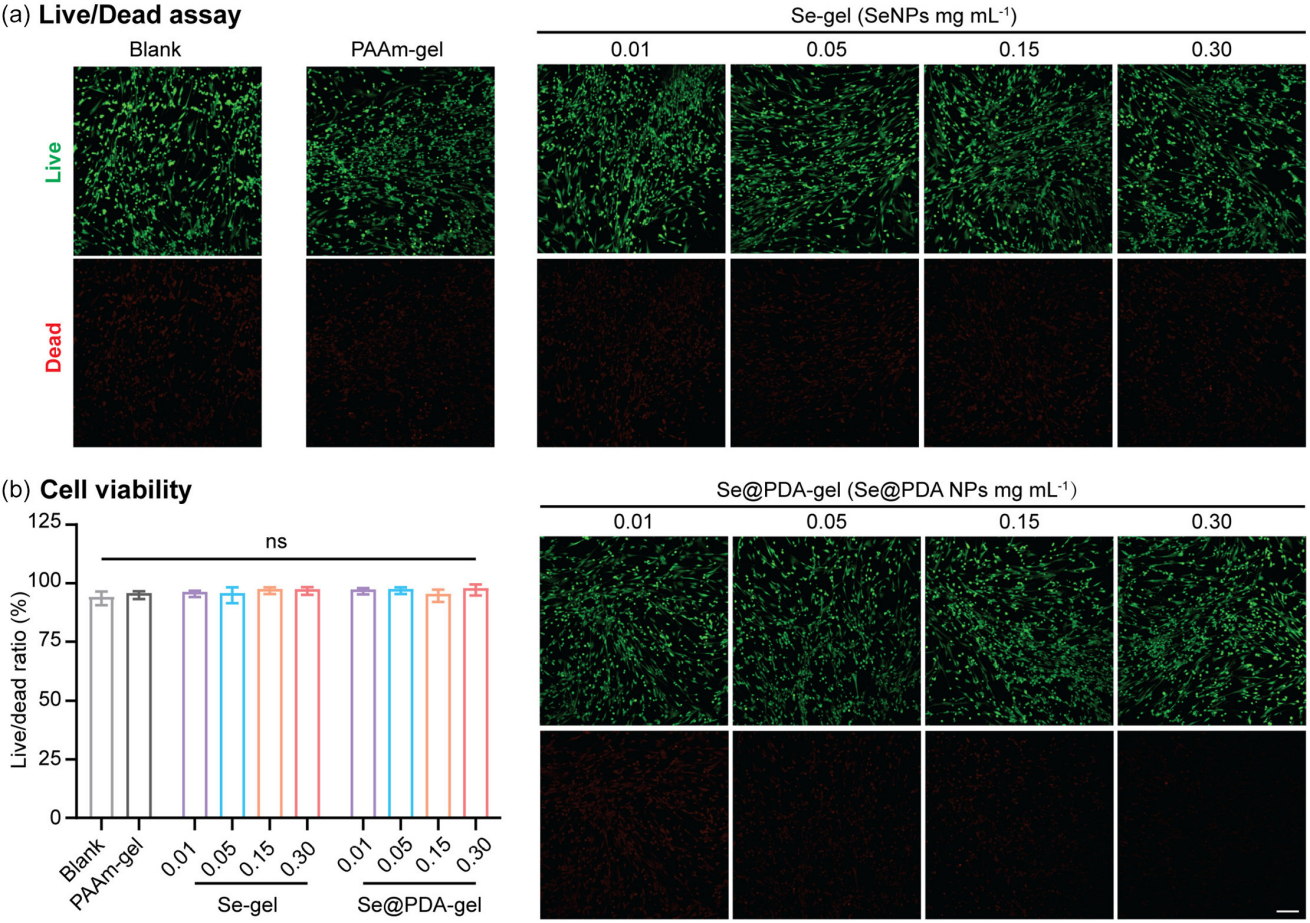
Cytotoxicity and cell viability evaluation of 3D PAAm hydrogels loaded with NPs. (a) Fluorescence microscope images of HCASMCs after 48 h of incubation with PAAm‐gel, Se‐gel, or Se@PDA‐gel with NPs loaded at various concentrations (0.01, 0.05, 0.15, or 0.3 mg mL^−1^). Cells stained with calcein (green) indicate live cells, and ethidium (red) indicate dead cells (scale bar 100 µm). (b) Corresponding cell viability analysis from Live/Dead assay. Statistical significance was calculated using one‐way ANOVA, ns = no significance. *n* = 5; error bars represent standard deviation.

### 2.6 In Vitro Cellular Effects

With its sustained NO‐generating capacity and high biocompatibility, Se@PDA‐gel holds great potential as a NO‐generating platform for therapeutic applications. To assess its therapeutic potential and suitability as a substrate for direct cell attachment, HUVECs were cultured on a thin layer of PAAm hydrogel‐coated glass coverslips containing Se@PDA NPs at concentrations of 0.05, 0.15, and 0.3 mg mL^−1^. HUVECs were selected due to their central role in angiogenesis and related cardiovascular processes [52]. After 48 h of culturing, a significant increase in cell number was observed on Se@PDA_0.3_‐gel (Figure 7a). Consistent with this growth enhancement, intracellular NO levels (quantified using DAF‐FM) were substantially elevated in the Se@PDA_0.3_‐gel group (Figure 7b). These findings suggest a positive correlation between NO generation and HUVEC proliferation on Se@PDA‐gel, emphasizing NO’s role in promoting HUVEC proliferation and highlighting the potential of Se@PDA‐gel for vascular repair and wound healing applications. To explore NO‐mediated immunomodulatory effects, IL‐10 expression, a key anti‐inflammatory cytokine, was evaluated via immunocytochemistry. Consistent with the elevated intracellular NO levels, IL‐10 expression was significantly upregulated in HUVECs cultured on Se@PDA_0.3_‐gel (Figure 7c). The increased IL‐10 expression suggested an enhanced anti‐inflammatory response that further emphasizes the therapeutic potential of the hydrogels. For instance, in atherosclerosis, a chronic inflammatory condition marked by the buildup of plaques in arterial walls, endothelial cells express adhesion molecules like ICAM‐1 [53] and VCAM‐1 [54], which attract immune cells. These immune cells, such as monocytes, differentiate into macrophages, ingest oxidized low‐density lipoprotein, and form foam cells on the vessel wall [55], exacerbating inflammation and accelerating plaque growth. IL‐10 counteracts these processes by reducing immune cell adhesion, migration, and macrophage activation, thereby limiting foam cell formation and helping stabilize or reduce plaques [56]. The elevated IL‐10 expression in HUVECs cultured on Se@PDA‐gel suggests its potential in anti‐inflammatory therapies, particularly for conditions like atherosclerosis. Confocal microscopy provided additional insights into the intracellular distribution of NO and IL‐10. As shown in Figure 7d, both NO and IL‐10 intensities were lower in PAAm‐gel compared to Se@PDA_0.3_‐gel. Surface colocalization analysis revealed that NO was distributed in both the nucleus and cytoplasm, while IL‐10 was primarily cytoplasmic. Merged images showed consistent alignment across fluorescent channels with the corresponding bright‐field images. While Se@PDA_0.3_‐gel demonstrated pronounced NO production and biological responses, restricted catalyst mass in the thin 2D configuration may limit overall NO availability. Consequently, subsequent studies were performed on 3D bulk hydrogels to further characterize NO‐mediated cellular effects.

**FIGURE 7:**
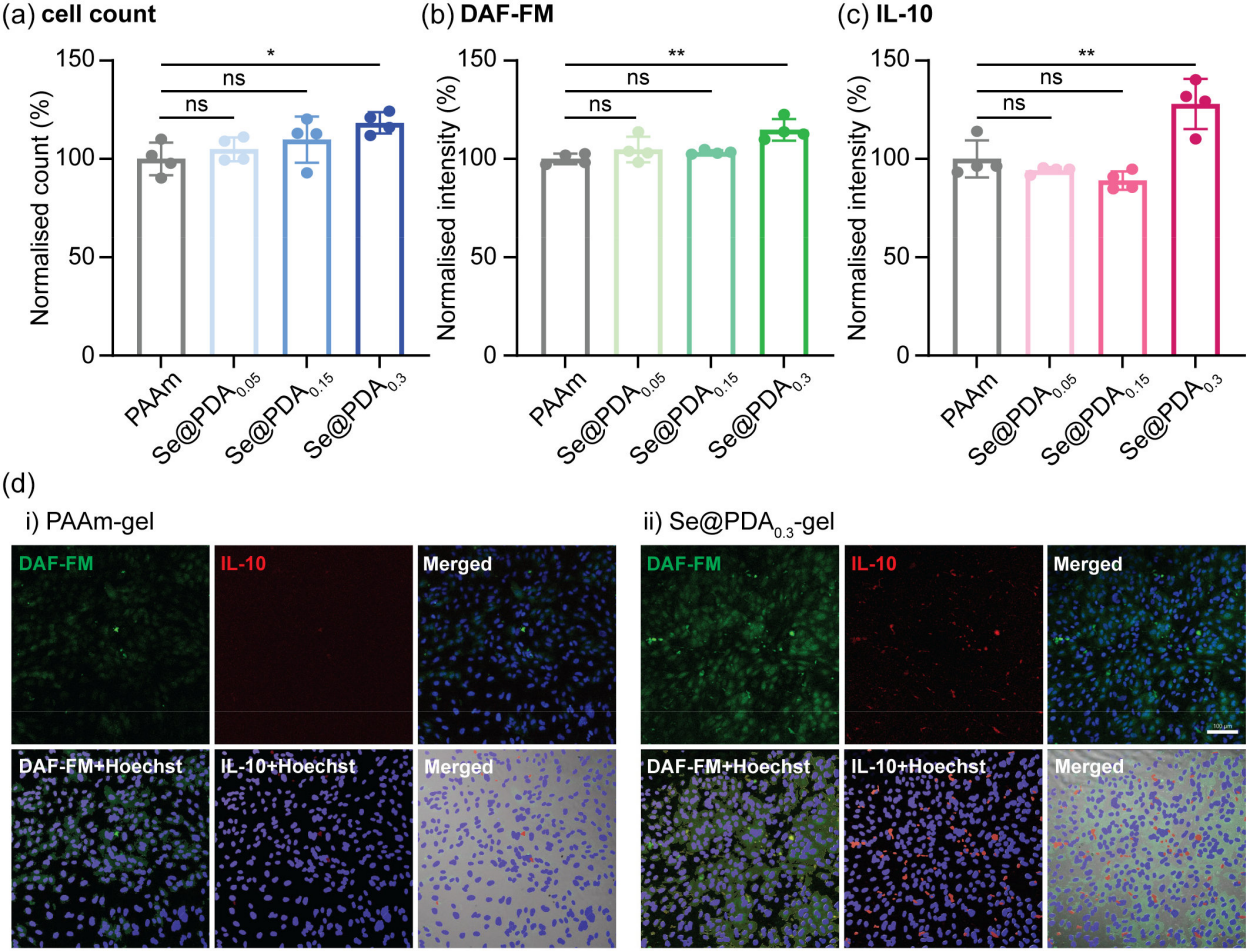
Effect of 2D Se@PDA‐gel coatings as direct cell substrates for HUVECs in vitro. Normalized (a) cell count, and fluorescence intensities of (b) DAF‐FM and (c) IL‐10. Fluorescence intensities were normalized to the corresponding cell number and expressed as a percentage of PAAm‐gel control. (d) Confocal image (top) and surface colocalization simulation (bottom) of HUVECs cultured on (i) PAAm‐gel and (ii) Se@PDA_0.3_‐gel, stained with DAF‐FM (green), Alexa Fluor 647‐conjugated anti‐human IL‐10 antibody (red), and Hoechst (blue) (scale bar 100 µm). Statistical significance was calculated using one‐way ANOVA, ns = no significance, *p < 0.05, **p < 0.01. *n* = 4; error bars represent standard deviation.

The cellular effects of 3D hydrogels were examined by incubating them indirectly with cells using 3D‐printed well inserts. After 48 h of incubation, intracellular NO levels in HUVECs, as measured by DAF‐FM, were upregulated at Se@PDA NP concentrations exceeding 0.05 mg mL^−1^ in a concentration‐dependent manner, with higher NP concentrations yielding increased catalytic NO production in vitro without the addition of NO donors (Figure 8a,d). Notably, while Se_0.3_‐gel displayed elevated NO levels compared with PAAm‐gel, the intensity was lower than that observed for Se@PDA_0.3_‐gel. This can be attributed to their lower stability in physiological conditions, as demonstrated in section 2.4, resulting in lower NO‐generating activity postincubation. IL‐10 expression was significantly increased with Se@PDA‐gel treatment, peaking at Se@PDA_0.05_‐gel (Figure 8b,d). The expression of the cytokine started to decline at Se@PDA NP concentrations of 0.15 mg mL^−1^ and decreased further at 0.3 mg mL^−1^, though levels remained above the PAAm‐gel control group. This trend likely reflects the concentration‐dependent effects of NO, which exhibits anti‐inflammatory properties at lower concentrations but may induce oxidative stress at higher levels, thereby attenuating its anti‐inflammatory effects [57].

**FIGURE 8:**
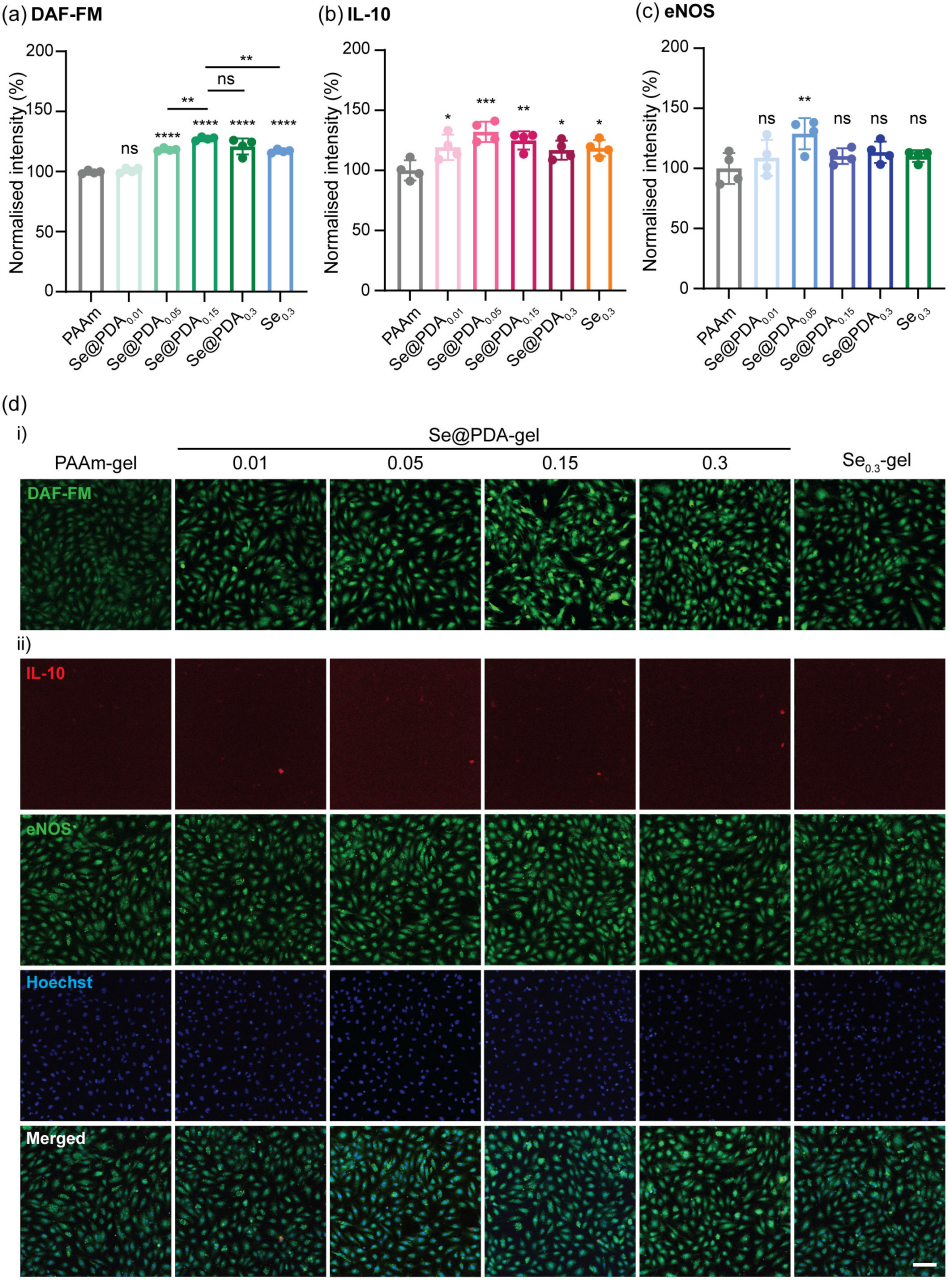
Se@PDA‐gel promotes in vitro NO production and anti‐inflammatory cytokine expression in HUVECs. Evaluation of fluorescence intensity of (a) DAF‐FM, (b) IL‐10, and (c) eNOS after 48 h of incubation with 3D hydrogels. Antibody expression intensities were normalized to the corresponding cell number and expressed as a percentage of PAAm‐gel control. (d) Confocal images of HUVECs treated with the hydrogels stained with (i) DAF‐FM (green) and (ii) Alexa Fluor 647‐conjugated anti‐human IL‐10 antibody (red), eNOS antibody (green), and Hoechst (blue), and merged views of IL‐10, eNOS and Hoechst (scale bar 100 µm). Statistical significance was calculated using one‐way ANOVA, ns = no significance, *p < 0.05, **p < 0.01, ***p < 0.001, ****p < 0.0001. *n* = 4; error bars represent standard deviation.

Endothelial nitric oxide synthase (eNOS) is one of the primary enzymes responsible for NO production. NO can exert both negative [58] and positive [59] feedback on eNOS. Specifically, excessive NO can interact with superoxide to form peroxynitrite (ONOO─), which damages eNOS and reduces its activity [60]. Conversely, NO can also upregulate eNOS expression in fetal intrapulmonary artery endothelial cells [59]. eNOS expression in HUVECs (Figure 8c,d) correlated with IL‐10 expression, with Se@PDA_0.05_‐gel identified as the optimal condition for inducing anti‐inflammatory effects while providing positive feedback to eNOS. Increased eNOS expression enhances NO production, which can improve vascular tone and reduce the risk of hypertension and other cardiovascular conditions [61, 62]. It has been reported that tetrahydrobiopterin (BH4), a crucial cofactor, plays a key role in regulating eNOS activity [63]. Adequate BH4 levels ensure efficient NO production by eNOS and prevent eNOS uncoupling, which otherwise leads to reactive oxygen species production instead of NO. Therefore, further studies are necessary to examine the effects of elevated eNOS expression in both physiological and pathological contexts. Confocal microscopy images of HUVECs (Figure 8d) illustrated an even distribution of eNOS throughout the cytoplasm and nucleus of HUVECs, with heightened expression of IL‐10 in certain cells.

In addition to HUVECs, HCASMCs are another crucial model for understanding various cardiovascular processes and diseases, such as atherosclerosis [64] and restenosis [65], both of which are closely linked to inflammation [66]. IL‐10 expression in HCASMCs (Figure 9a,d) followed a similar pattern to that of HUVECs (Figure 8b), with the highest levels observed at Se@PDA_0.05_‐gel treatment, highlighting the concentration‐dependent anti‐inflammatory effects of hydrogels. While eNOS is predominantly expressed in endothelial cells, it is also present in platelets, cardiac myocytes [67], fibroblast cells [68], and vascular smooth muscle cells [69]. However, unlike the increased eNOS expression observed in HUVECs, no significant changes in eNOS expression were detected in HCASMCs (Figure 9b,d), indicating a cell‐type‐specific response to NO and suggesting that distinct pathways may govern NO‐eNOS interactions in smooth muscle cells. Further investigation is required to elucidate these mechanisms. In contrast to the NO‐induced proliferation observed in HUVECs, the cell counts of HCASMCs treated with hydrogels showed no significant increase (Figure 9c), confirming NO’s regulatory effect on SMC proliferation [70]. This effect is particularly relevant in cardiovascular contexts, where NO helps regulate vascular tone and prevents abnormal thickening of vessel walls, a process implicated in atherosclerosis and restenosis. Cell counts were correlated with cytotoxicity results (Figure 6), showing no toxic effects or reduction in cell viability following hydrogel treatment. Confocal microscopy images (Figure 9d) revealed the distribution of IL‐10 and eNOS in both the cytoplasm and nucleus of HCASMCs. Overall, the elevated IL‐10 expression in both HUVECs and HCASMCs highlights the anti‐inflammatory potential of Se@PDA‐gel, highlighting its promise for treating inflammation‐associated cardiovascular diseases.

**FIGURE 9:**
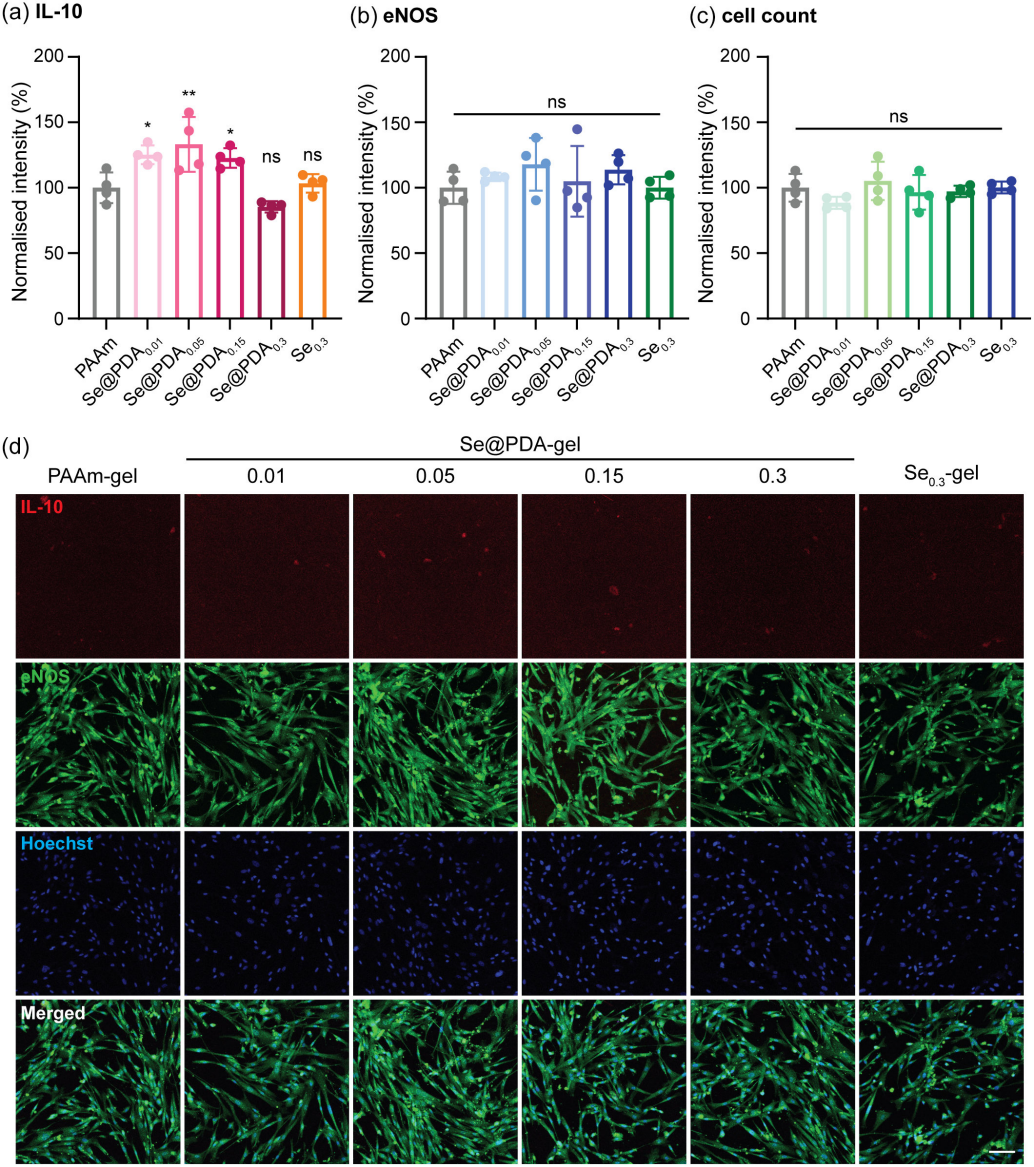
3D Se@PDA‐gel promotes anti‐inflammatory cytokine expression in HCASMCs. Evaluation of (a) IL‐10, (b) eNOS, and (c) cell number of HCASMCs after 48 h of incubation with 3D hydrogels. Antibody expression intensities were normalized to the corresponding cell number and expressed as a percentage of PAAm‐gel control. (d) Confocal images of HCASMCs treated with the hydrogels stained with Alexa Fluor 647 conjugated anti‐human IL‐10 antibody (red), eNOS antibody (green) and Hoechst (blue) (scale bar 100 µm). Statistical significance was calculated using one‐way ANOVA, ns = no significance, *p < 0.05, **p < 0.01, ***p < 0.001, ****p < 0.0001. *n* = 4; error bars represent standard deviation.

Given the upregulation of HUVEC proliferation in response to NO, we further investigated the potential of Se@PDA‐gel for wound healing by performing immunostaining of α‐SMA on NIH 3T3 fibroblasts. α‐SMA is a protein primarily found in smooth muscle cells, where it plays a crucial role in contractile function. However, α‐SMA is also expressed in other circumstances, such as fibroblast‐myofibroblast transition (FMT), where activated fibroblasts transition into myofibroblasts, a process critical for tissue regeneration and wound healing [71, 72]. As shown in Figure 10a, α‐SMA expression was significantly elevated in fibroblasts cultured with Se@PDA_0.15_‐gel, with expression intensity increasing further in the Se@PDA_0.3_‐gel group. The upregulation of α‐SMA suggests that the fibroblasts underwent transitions to a more contractile phenotype resembling FMT, which is essential for effective wound healing and tissue repair. On the other hand, Se_0.3_‐gel exhibited no significant difference in α‐SMA expression compared to the PAAm‐gel control, which can be attributed to its lower stability in physiological conditions. In the context of wound healing, eNOS deficiency has been associated with impaired wound healing and angiogenesis in mice [73], while therapeutic delivery of the eNOS gene has been shown to enhance wound healing in compromised models [74]. To assess the effect of Se@PDA‐gel on eNOS expression, we measured eNOS expression in NIH 3T3 cells after 48 h of incubation with the hydrogels. A significant increase in eNOS expression was observed in cells treated with Se@PDA_0.3_‐gel (Figure 10b), suggesting a positive correlation between Se@PDA NPs and eNOS upregulation. However, the exact pathways involved remain unclear, warranting further investigation into their physiological and pathological implications. Confocal microscopy images of fibroblasts (Figure 10c) show the distribution of α‐SMA and eNOS in both the cytoplasm and nucleus. The elevated expression of α‐SMA and eNOS highlights the potential of Se@PDA‐gel in promoting wound healing.

**FIGURE 10:**
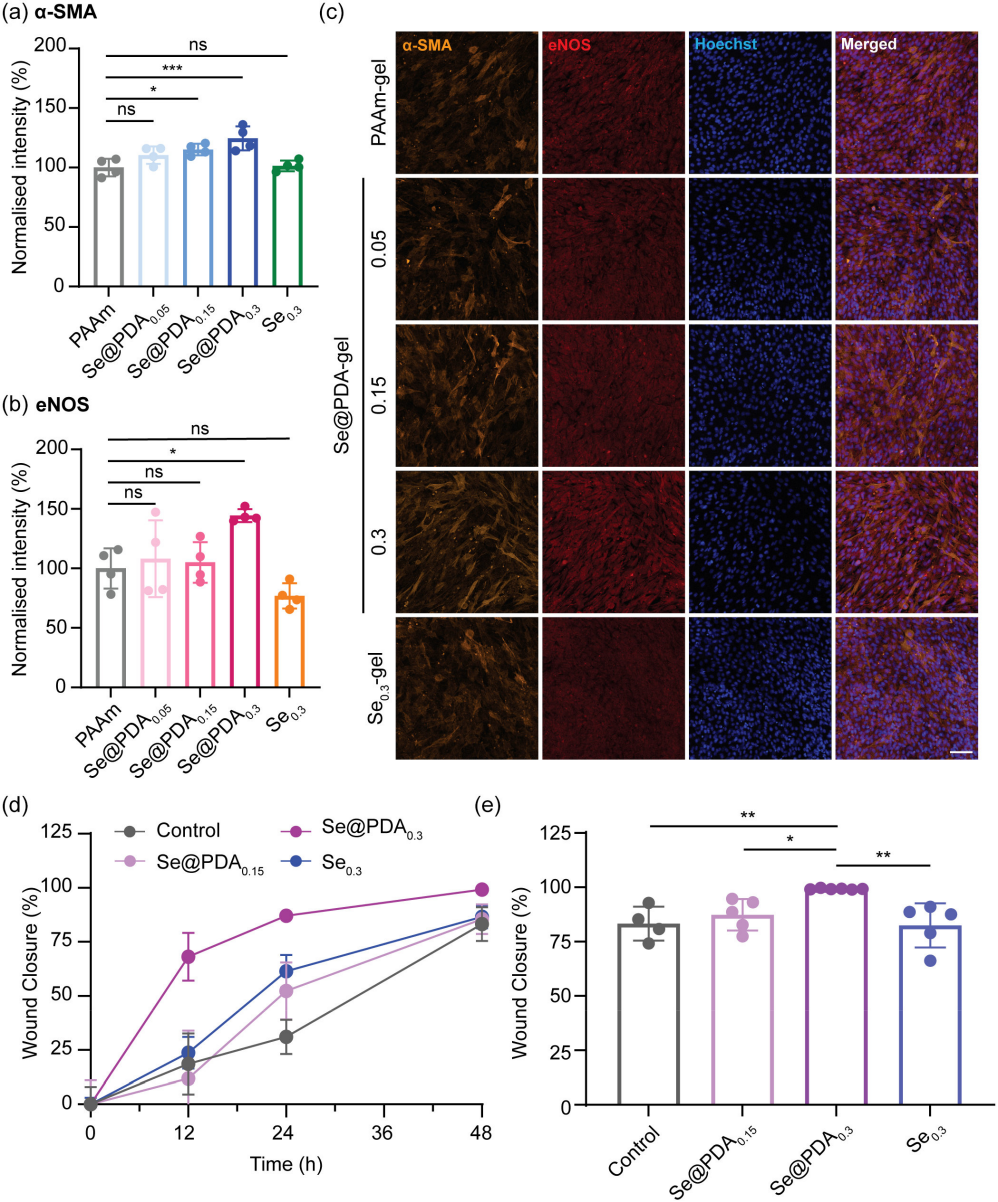
Immunocytochemistry analysis of the 3D hydrogels on NIH 3T3 cells in vitro. Evaluation of (a) α‐SMA and (b) eNOS expression in NIH 3T3 cells after 48 h of incubation with 3D hydrogels. Antibody expression intensities were normalized to the corresponding cell number and expressed as a percentage of PAAm‐gel control. (c) Confocal images of NIH 3T3 cells treated with the hydrogels stained with α‐SMA antibody (orange), eNOS antibody (red), and Hoechst (blue) (scale bar 100 µm). (d) Quantitative analysis of NIH 3T3 fibroblast scratch wound closure over 48 h. (e) Wound closure percentage at 48 h. Statistical significance was calculated using one‐way ANOVA, ns = no significance, *p < 0.05, **p < 0.01, ***p < 0.001. *n* = 4; error bars represent standard deviation.

Additionally, an in vitro wound healing assay was performed to evaluate the effects of Se@PDA hydrogels on fibroblast migration and wound closure (Figure S12). A linear scratch wound was generated on a confluent monolayer of NIH 3T3 cells, and wound closure was monitored over 48 h. As shown in Figure 10d, treatment with Se@PDA hydrogels markedly accelerated wound closure compared with the untreated control group in a concentration‐dependent manner. Among all groups, Se@PDA_0.3_‐gel demonstrated the greatest enhancement in cell migratory activities, particularly during the early phase of wound healing, achieving approximately 87% wound closure within the first 24 h. In comparison, the untreated control group displayed substantially slower wound closure kinetics, with 31.1% closure after 24 h. Se@PDA_0.15_‐gel also promoted fibroblast migration, resulting in 52.3% wound closure, suggesting a dose‐dependent effect of Se@PDA nanoparticles. After 48 h, both treated and untreated groups exhibited over 80% wound closure, while near‐complete wound closure was observed for the Se@PDA_0.3_‐gel group (Figure 10e). The markedly accelerated in vitro wound healing behavior induced by Se@PDA_0.3_‐gel is consistent with the upregulation of α‐SMA and FMT tendency observed in immunofluorescence studies, further corroborating the ability of Se@PDA hydrogels to promote fibroblast activation and migratory responses that are critical for effective wound repair.

## 3 Conclusions

Sustained NO delivery presents a challenge due to NO’s short half‐life. Catalytic strategies that leverage enzyme‐mimicking nanomaterials have therefore gained increasing attention as they enable continuous in situ NO generation from endogenous donors. This study demonstrated that Se@PDA‐incorporated hydrogels exhibited stable NO‐generating capacity, with tunable NO generation achieved by adjusting the concentration of Se@PDA NPs loaded into the hydrogels. The high biocompatibility and versatile nature of hydrogels broaden their therapeutic potential. When HUVECs were cultured on Se@PDA‐gel without adding NO donors, elevated NO levels were detected by DAF‐FM, confirming the NO‐generating abilities in vitro. Furthermore, elevated IL‐10 expression in HUVECs and HCASMCs suggests their potential for anti‐inflammatory applications, while increased α‐SMA expression in 3T3 cells indicates their potential in wound healing. Future research should focus on elucidating the mechanisms of positive and negative feedback of NO on eNOS, assessing the system’s therapeutic effect in vivo, and thereby advancing the clinical applications of this promising NO‐generating system. In conditions where endogenous RSNO levels are insufficient to sustain effective NO generation, the codelivery of Se@PDA NPs with exogenous NO donors may represent a viable strategy to maintain therapeutic NO levels. While the present study demonstrates the promising NO‐generating capability and favorable biological responses of the Se@PDA‐gel system in vitro, further evaluation in relevant in vivo models will be essential to fully assess its therapeutic efficacy and translational applicability.

## 4 Experimental Section

### 4.1 Materials

Acrylamide (AAm), *N*,*N′‐*methylenebisacrylamide (bis‐AAm), *N*,*N*,*N′*,*N′‐*tetramethyl ethylenediamine (TEMED), ammonium persulfate (APS), sodium selenite (Na_2_SeO_3_), ascorbic acid (C_6_H_8_O_6_), polyvinyl alcohol (PVA; Mw 90–98 kDa), dopamine hydrochloride, glutathione reduced (GSH), Griess reagent (modified), phosphate buffered saline (PBS), Tris hydrochloride (Tris‐HCl), and (3‐aminopropyl) triethoxysilane (APTS) were purchased from Sigma–Aldrich. Potassium nitrite (KNO_2_, ≥ 96%), sodium chloride (NaCl), potassium iodide (KI), sulfuric acid (H_2_SO_4_), bovine serum albumin (BSA), and glutaraldehyde were purchased from Chem‐Supply. *S*‐Nitrosoglutathione (GSNO) was purchased from MedChemExpress. *S*‐Nitroso*‐N*‐Acetyl‐DL‐Penicillamine (SNAP) was purchased from Cayman Chemical. Paraformaldehyde (PFA, 16% w/v) and Triton X‐‍100 were obtained from Thermo Scientific. Hydrazine hydrate was acquired from Acros Organics, and sodium periodate was purchased from Univar. Ultrapure water (18.2 MΩ·cm resistance) provided by arium mini Sartorius was used throughout the experiments. All reagents were used without further purification.

### 4.2 Synthesis of SeNPs and Se@PDA NPs

SeNPs and Se@PDA NPs were synthesized following our previous studies [26, 27]. To synthesize SeNPs, 5 mL of sodium selenite (100 mM in ultrapure water) and 5 mL of PVA (1 mg mL^−1^ in ultrapure water) were mixed at 800 RPM at room temperature. Then, 10 mL of ascorbic acid (250 mM in ultrapure water) was introduced into the above mixture at a flow rate of 4 mL min^−1^, after which the solution was stirred for another 30 min under the same conditions. Then, the resulting SeNPs were washed 3 times by centrifugation at 9500 RPM for 15 min and stored in ultrapure water at 2.5 mg mL^−1^. To synthesize Se@PDA NPs, 1.6 mL of SeNPs (2.5 mg mL^−1^) was mixed with 13.8 mL of Tris‐HCl (0.1 M, pH 8.0) and 4 mL of ethanol. After 5 min of stirring at 21 °C and 400 RPM, 0.6 mL of dopamine‐HCl (10 mg mL^−1^ in ultrapure water) was added. The mixture was stirred for another 2 h at 1000 RPM, followed by washing twice with ultrapure water via centrifugation at 10 000 RPM for 15 min. The Se@PDA NPs were stored in ultrapure water at 1.0 mg mL^−1^ at 4 °C for future use.

### 4.3 Synthesis of Hydrogels

PAAm hydrogels were prepared following previously described methods with modifications [28, 75]. To synthesize 3D bulk hydrogels, 30 µL of AAm monomer (40 wt%), 25 µL of the cross‐linking agent bis‐AAm (2 wt%), and 45 µL of SeNPs or Se@PDA NPs colloidal solution were placed in a 96‐well plate. The final concentrations of SeNPs or Se@PDA NPs in the 100 µL hydrogels were adjusted to 0.01, 0.05, 0.15, or 0.3 mg mL^−1^. Then, 2 µL of the catalyst TEMED (10 %) and 3 µL of the initiator APS (10 wt%) were added, followed by rapid pipetting to ensure a homogeneous mixture. These hydrogels were denoted as PAAm‐gel, Se_*x*_‐gel, and Se@PDA_*x*_‐gel, respectively, where ‘*x*’ represents the concentration of SeNPs or Se@PDA NPs (in mg mL^−1^) in 100 µL of hydrogel. Note that for PAAm‐gel, 45 µL of ultrapure water was added in place of the SeNPs or Se@PDA NPs suspension. 2D thin‐film hydrogel coatings were synthesized on 18 mm glass coverslips using the same concentrations of AAm, bis‐AAm, Se@PDA NPs, TEMED, and APS as in the 3D bulk hydrogels, but with a reduced total volume of 20 µL. The glass coverslips were activated and pretreated with APTS (0.5%) and glutaraldehyde (0.5%) before gelation. The hydrogel mixture was placed onto the treated coverslip, and a circular coverslip was gently placed over it to generate an even coating. The setup was left undisturbed for 45 min to allow for complete polymerization. The surface of the 2D hydrogel coating was treated with hydrazine hydrate and acetic acid (5%) for protein conjugation, and the protein solution was made with sodium periodate (3.5 mg mL^−1^) and fibronectin (25 μg mL^−1^) in PBS to generate free aldehydes for cell attachment. All the chemicals were prepared in ultrapure water. The 2D hydrogels were used exclusively as a substrate for direct attachment and growth of HUVECs in Section 2.6, and the 3D hydrogels were employed for all other experiments and placed in 3D‐printed inserts submerged in the cell medium to incubate with cells indirectly.

### 4.4 Characterization

SeNPs and Se@PDA NPs were characterized using transmission electron microscopy (JEOL F200 TEM) operated at an accelerating beam voltage of 200 kV. The compositions of SeNPs and Se@PDA NPs were characterized by elemental mapping using TEM operating in HAADF–STEM mode. Field emission scanning electron microscopy (FESEM, FEI Nova NanoSEM 230) was used to characterize both NPs and hydrogels at an acceleration voltage of 10 kV and a spot size of 3. NP samples were prepared by pipetting 10 μL of the NP solution (2 mg mL^−1^ in ultrapure water) onto silica wafers and drying them overnight at room temperature. Hydrogel samples were prepared by freezing using liquid nitrogen, followed by overnight freeze‐drying. To enhance the electron signal, all samples were coated with a 15 nm platinum layer using a Leica ACE600 sputter coater. The elemental composition of hydrogels was investigated using field emission scanning electron microscopy (FEI Nova NanoSEM 450) fitted with energy dispersive spectroscopy (EDS, Bruker SDD‐EDS) to confirm successful loading of SeNPs or Se@PDA NPs. For EDS analysis, the samples were coated with a 15 nm carbon layer using a Safematic carbon coater. The composition and chemical structure of the hydrogels were characterized using Raman spectroscopy, FT‐IR, XRD, and XPS. Raman spectra were collected via a Raman spectrometer (inVia Raman microscope, Renishaw) with a 532 nm laser source, 1% laser power, 1800 grooves mm^−1^ grating, and an acquisition time of 30 s per sample. FT‐IR spectra were recorded in transmission mode using a PerkinElmer Spectrum 10, with a resolution of 1 cm^−1^ and 20 accumulated scans. The crystal structure of the hydrogels was investigated using (PANalytical Empyrean I) at 40 kV and 40 mA with a Cu‐Kα radiation source. The elemental composition and chemical structure of the hydrogels were further analyzed by XPS (Thermo ESCALAB250Xi) with a monochromated Al Kα X‐ray radiation source (energy 1486.68 eV) operating at 120 W. The pass energies for survey scans and region scans were 100 and 20 eV, respectively. Se_0.15_‐gel and Se@PDA_0.15_‐gel were used for all the characterizations.

### 4.5 Mechanical Properties

The physical and mechanical properties of hydrogels were investigated through water content, swelling ratio, and compression tests. The water content of the hydrogels was measured by calculating the mass differences before and after freeze‐drying. The swelling ratio of both fresh and freeze‐dried hydrogels was calculated using the following equation

$$\text{Swelling ratio}=\;(W_{s}-W_{i})\;/W_{i}\times 100\%$$

where *W*_*i*_ and *W*_s_ are the hydrogel weights before and after incubation with ultrapure water, respectively. The compression test was performed using a motorized compression test stand (ESM303, Mark‐10). The hydrogels for compression tests were synthesized in a 24‐well plate with a total volume of 1 mL. The hydrogel samples were compressed with a compression rate of 1.5 mm min^−1^. The engineering strain under compression was calculated as:

Engineering strain (ε)= Δh/h

where *h* is the original height of the hydrogel and Δh is the difference between the deformed height and the original height. The engineering stress was calculated as:

Engineering stress (σ) = F/A

where *F* is the force applied on the hydrogel and *A* is the top surface area of the hydrogel. True strain and stress were calculated using the two equations:

$$\text{True strain }(\varepsilon_{t})=\text{− }\ln\;(1-\varepsilon )$$

$$\text{True stress }(\sigma_{t})=\sigma \;(1-\varepsilon )$$

### 4.6 Stability

The stability of hydrogels was investigated through 5‐day recycling and long‐term storage tests. For the 5‐day recycling test, the hydrogels were incubated with NO donors (50 µM) and GSH (1 mM) in PBS buffer, and the cumulative NO generation was measured using the Griess assay after 15, 30, and 60 min. After the first cycle, the hydrogels were washed using ultrapure water and stored in PBS overnight at room temperature. On the second day, the same incubation conditions were applied, followed by another washing process. This process was repeated for a total of 5 days.

The long‐term stability of the hydrogels was evaluated using two methods. First, the hydrogels were stored in PBS buffer at 4 °C for 2 months, after which their 30 min cumulative NO generation was measured using the Griess assay. Second, the hydrogels were freeze‐dried and rehydrated after 2 months, followed by a 30‐min cumulative NO generation measurement using the Griess assay.

### 4.7 Se Leaching

SeNPs or Se@PDA NPs leaching from hydrogels under physiological conditions were measured by incubating three pieces of 3D bulk hydrogels (Se_0.3_‐gels or Se@PDA_0.3_‐gels) in 10 mL of PBS at 37 °C and 300 RPM for 6, 12, 24 h, and 7 days. The supernatant was analyzed by inductively coupled plasma mass spectrometry (Perkin Elmer, Nexion 5000). The instrument settings included: ICP RF power: 1200–1500 W, vacuum pressure: <8 × 10^−6 ^torr, pulse stage voltage: 800–1500 V, analog stage voltage: −(1700–2300) volt, plasma gas flow: 15–17 L min^−1^, auxiliary gas flow: 1–2 L min^−1^, nebulizer gas flow: 0.85–1.2 L min^−1^, lense voltage: 4–12 V, and cell gas: He, NH_3_, O_2_, CH_4_. The instrument was calibrated with a freshly prepared series of standards from a stock standard solution, which included the required element (at least 4 points including a blank) before starting analysis of a batch.

### 4.8 NO Generation

For real‐time monitoring of NO generation, a free‐radical analyzer (TBR4100, World Precision Instruments) equipped with a NO‐sensitive probe (ISO‐NOP, World Precision Instruments) was used. The NO electrode was first immersed in 3.95 mL of PBS (10 mM, pH 7.4) with GSH (1 mM) and one piece of 3D hydrogel at 37 °C in the dark under constant stirring (400 RPM) until a stable baseline was reached. Then, 50 µL of NO donors (GSNO or SNAP) was added to the suspension to a final concentration of 10 µM. The changes in current were recorded and converted into NO concentrations using a calibration curve. To assess sustained NO generation from the hydrogels, the same procedure was repeated for five sequential NO donor additions, allowing the signal to return to baseline between additions.

The cumulative NO generation was quantified using the Griess assay. One piece of hydrogel was incubated at 37 °C in the dark in 1 mL of PBS buffer containing NO donors (50 µM) and GSH (1 mM). After incubation, 75 µL of the supernatant was mixed with an equal volume of Griess reagent (40 mg mL^−1^ in PBS) for 15 min in the dark. The absorbance was then measured at 546 nm using a SpectraMax M5 microplate reader. The concentration of NO was then calculated based on a calibration curve.

### 4.9 Biocompatibility

The biocompatibility of hydrogels was investigated using HCASMCs from Cell Applications. HCASMCs were cultured in smooth muscle cell growth medium and sub‐cultured when they reached a near‐confluent state. For the cytotoxicity test, HCASMCs were seeded onto 24‐well plates at a density of 20 000 cells 500 µL^−1 ^well^−1^ and incubated for 24 h at 37 °C in a 5% CO_2_‐humidified incubator. Then, the hydrogels (sterilized under a UV lamp for 30 min prior to use) were placed into 3D‐printed inserts and incubated in the medium with cells. After a 48 h incubation, the 3D‐printed inserts containing the hydrogels were removed, and cells were washed with PBS once. Their viability was quantified using the Live/Dead assay (L3224, Thermo Fisher), where cell medium containing calcein AM (2 μM) and ethidium homodimer‐1 (4 μM) was added to each sample and incubated at 37 °C in a 5% CO_2_ incubator for 45 min. Then, the samples were washed with PBS once and imaged using a Zeiss LSM 800 confocal microscope, and the numbers of live and dead cells were counted using Imaris software (Oxford Instruments). Cell viability was calculated based on the following equation

$$\mathrm{Cell}\text{ Viability }\%=\frac{\mathrm{Live}\text{ Cells}}{\mathrm{Live}\text{ Cells}+\mathrm{Dead}\text{ Cells}}\times 100\%$$

### 4.10 Cell Staining

Fluorescence and immunofluorescence staining were performed to characterize intracellular NO production and cell behaviors. Cells were plated in 12‐ or 24‐well plates until ~50% confluent before incubation with hydrogels. After 48 h of incubation, the hydrogels were removed from the well, and the cells were stained with intracellular NO fluorescent probe DAF‐FM diacetate (D23842, Thermo Fisher) at 5 µM and nuclear counterstain Hoechst 33342 (62249, Thermo Fisher) at 4 µM. For immunofluorescence staining, cells were fixed with PFA (4%) for 1 h, permeabilized with Triton X‐100 (0.2%) for 30 min, and blocked with BSA (2.5%) for 30 min. Cells were then incubated with primary antibodies, followed by secondary antibodies for 2 h minimum. Samples were washed 3 times between steps. Primary antibodies α‐SMA (A5228, Sigma–Aldrich) and eNOS (PA3‐031A, Thermo Fisher) were stained at 1:300, and Alexa Fluor 647‐conjugated anti‐human IL‐10 (501412, BioLegend) was stained at 1:200. Secondary antibodies Alexa Fluor 555 conjugated donkey anti‐mouse IgG (H + L) (A‐31570, Thermo Fisher), Alexa Fluor 488‐conjugated donkey anti‐rabbit IgG (H + L) (A‐21206, Thermo Fisher), and CF 647 conjugated goat anti‐rabbit IgG (H + L) (SAB4600184, Sigma–Aldrich) were stained at 1:400. Confocal microscopy was performed on Zeiss LSM 800, and images acquired were processed using Imaris software to quantify fluorescence intensity and localization.

### 4.11 Wound Healing Assay

Cell migration and wound healing behavior were evaluated using a scratch assay. Mouse fibroblasts NIH 3T3 were seeded in 24‐well plates at a density of 40 000 cells 500 µL^−1 ^well^−1^ and allowed to stabilize for 24 h. Cells were then incubated with sterilized hydrogels for 24 h until reaching 90%–100% confluency. A linear scratch was generated using a sterile 200 µL pipette tip, followed by gentle washing with DPBS to remove detached cells and debris. Fresh medium was added to the cells, and images were acquired at 0, 12, 24, and 48 h using an optical microscope. Wound areas were quantified using the Wound Healing Size Tool plugin in ImageJ (NIH). The original plugin was modified to calculate the summed area of all segmented wound regions in each image, enabling improved quantification of fragmented or discontinuous wound gaps. Wound closure was calculated relative to the initial wound area, where *A*_0_ represents the initial wound area and *A*_t_ represents the wound area at the indicated time point.

$$\mathrm{Wound}\;\mathrm{closure}\;\%=\frac{A_{0}-A_{t}}{A_{0}}\times 100\%$$

### 4.12 Statistical Analysis

NO levels, cell viabilities, and fluorescent intensities are presented as mean ± standard deviation. Sample sizes (*n* = 3 for NO levels, *n* = 5 for cell viabilities, and *n* = 4 for fluorescence intensities) are specified in figure captions. Statistical analyses were performed using GraphPad Prism 10 software. One‐way ANOVA followed by Tukey’s post hoc test (*α* = 0.05) and two‐way ANOVA with Tukey’s multiple comparison test (*α* = 0.05) were used to assess statistical significance. Significant differences between the groups were expressed as: ns = no significance, *p < 0.05, **p < 0.01, ***p < 0.001, and ****p < 0.0001.

## Funding

This study was supported by National Health and Medical Research Council Investigator Grant (2043381).

## Conflicts of Interest

The authors declare no conflicts of interest.

## Supporting information

Supplementary Material

## Data Availability

The data that support the findings of this study are available from the corresponding author upon reasonable request.
