# Supplementary material for: Catalytic Nitric Oxide‐Generating Selenium Nanoparticle‐Loaded Hydrogels With Anti‐Inflammatory and Pro‐Wound‐Healing Properties
Source: Small Sci. 2026 Sep 21;6(9):e70409. doi: 10.1002/smsc.70409 (PMC13618612; doi:10.1002/smsc.70409)
Supplement: Supplementary file 1 [file SMSC-6-e70409-s001.pdf]

Supporting Information

Catalytic nitric oxide-generating selenium nanoparticle-loaded hydrogels with anti-Inflammatory and pro-wound-healing properties

Shu Geng,†[a] Kang Lin,†[a] Qingqing Fan,[a] Yulong Sun,[a] Federico Mazur,[a] and Rona Chandrawati [a,*]

[a] School of Chemical Engineering and Australian Centre for Nanomedicine (ACN), The University of New South Wales (UNSW Sydney), Sydney, NSW 2052, Australia

† These authors contributed equally to this work

E-mail: rona.chandrawati@unsw.edu.au

Table S1. Assignment of Raman shifts

Reference shift (cm^{-1})	This work (cm^{-1})	Assigned bond
481[1]	489	Skeletal bending
644[1]	649	CH_2 torsion
773[1]	777	C-H wagging
844[1]	844	CH_2 twisting
1113[1]	1118	Polymer skeletal vibration
1211[2]	1205	NH_2 wagging
1327[2]	1329	C-H bending
1428[2]	1422	C-N stretching
1459[2]	1455	CH_2 bending
1618[2]	1612	NH_2 bending
1660[2]	1662	C=O stretching (amide)

Table S2. Assignment of FT-IR shifts

Reference shift (cm^{-1})	This work (cm^{-1})	Assigned bond
3326[3]	3338	N-H stretching
3168[3]	3194	N-H stretching
2924[4]	2920	CH_2 stretching
1664[5]	1639	C=O (amide) stretching
1610[3]	1600	N-H bending (amide I)
1452[5]	1442	CH_2 in-plane vibration
1420[5]	1413	C-N stretching
1320[6]	1313	-NH stretching
1188[6]	1184	-NH deformation
1125[5]	1110	NH_2 in-plane rocking

Table S3. Assignment of XPS peaks

Peak		Assigned bond	Binding energy (eV) and area %		
			Se-gel	Se@PDA-gel	PAAm-gel
O1s	O1s A	C=O	531.24 eV (90.38 %)	531.20 eV (85.61 %)	531.30 eV (97.60 %)
	O1s B	C-O	532.88 eV (9.62 %)	532.96 eV (14.39 %)	532.72 eV (12.4 %)
N1s	N1s A	N-C=O	399.51 eV (100 %)	399.47 eV (85.21 %)	399.57 eV (100 %)
	N1s B	R_2-NH (pyrrole)		400.61 eV (4.55 %)	
	N1s C	R-NH_2 (amine)		402.08 eV (10.24 %)	
C1s	C1s A	C-C/C-H[7]	284.8 eV (67.97 %)	284.8 eV (60.26 %)	284.80 eV (68.90 %)
	C1s B	C-O[7]	286.12 eV (5.31 %)	286.19 eV (15.49 %)	286.12 eV (6.23 %)
	C1s C	N-C=O[7]	287.91 eV (26.73 %)	287.89 eV (24.26 %)	287.92 eV (24.87 %)

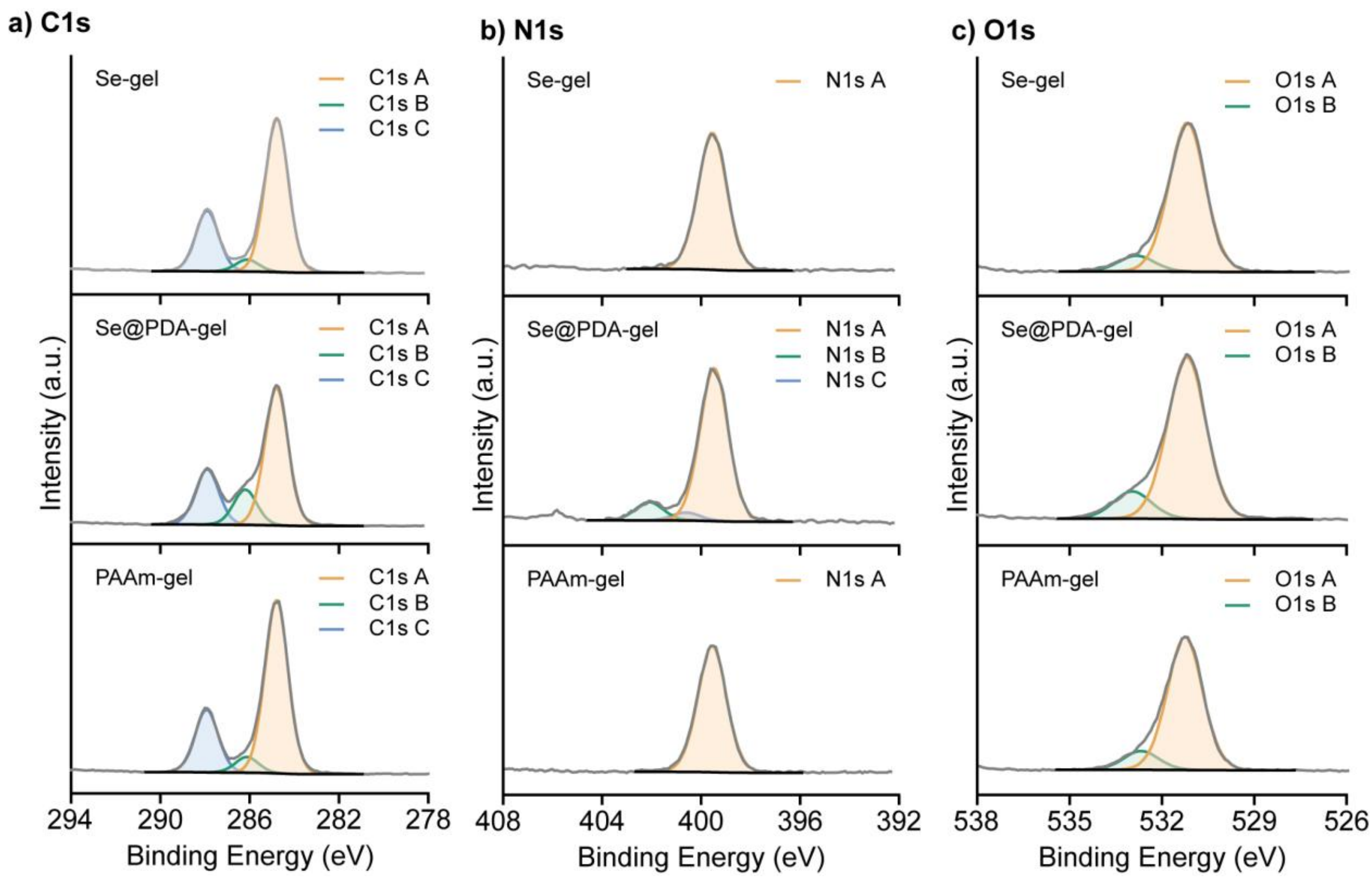

Figure S1. High-resolution XPS spectra of a) C1s, b) N1s, and c) O1s for Se-gel, Se@PDA-gel, and PAAm-gel.

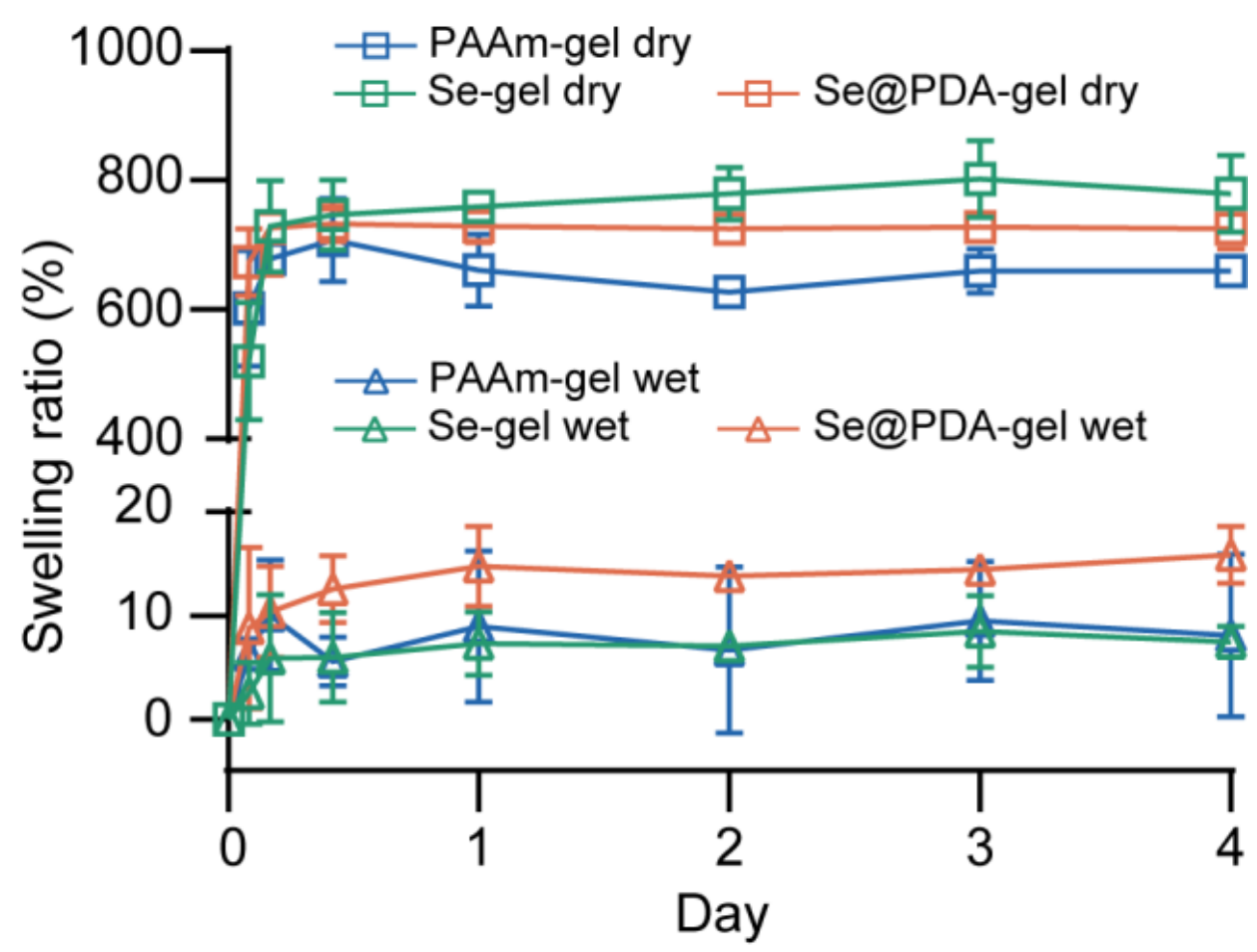

Figure S2. Swelling ratio of PAAm-gel, Se-gel, and Se@PDA gel at room temperature. n=3; error bars represent standard deviation.

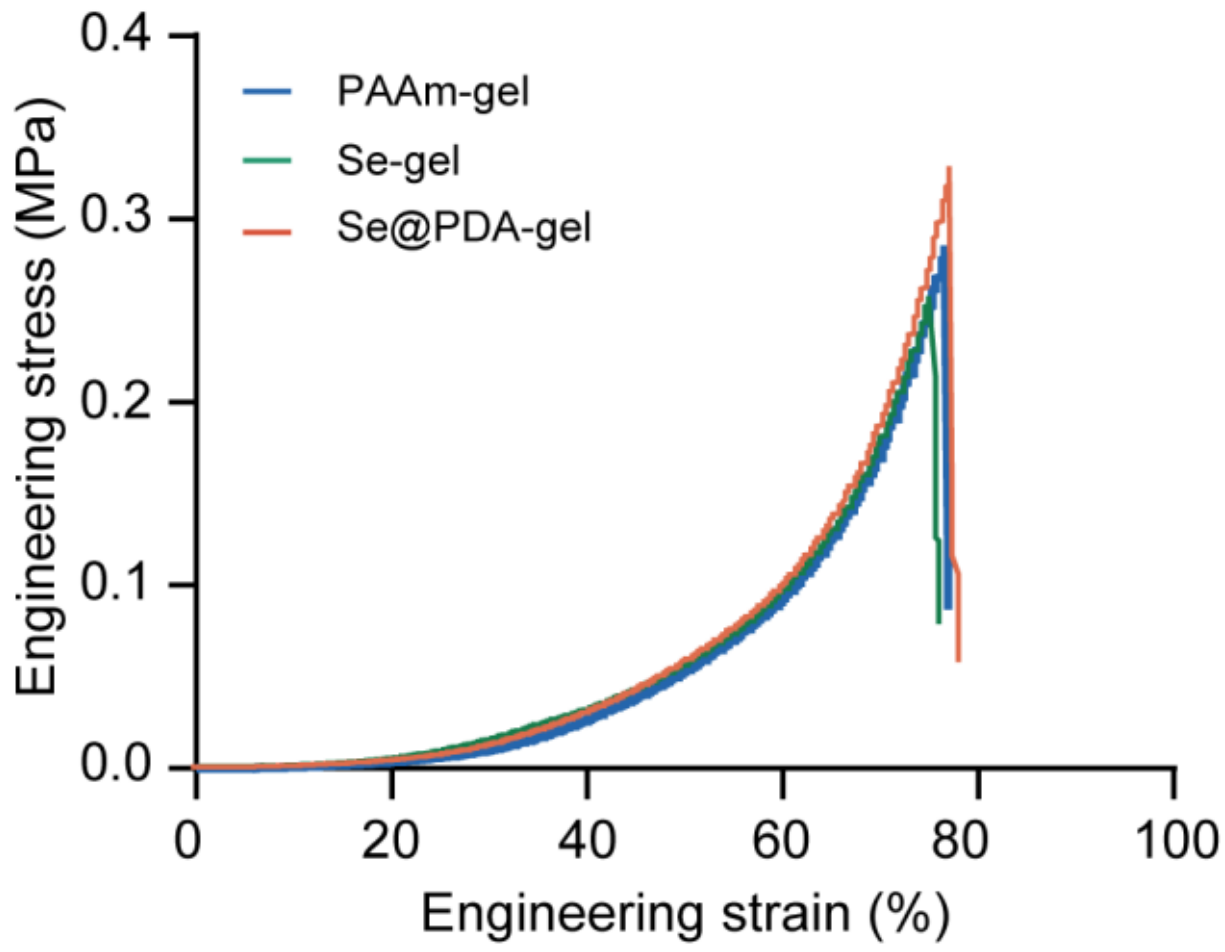

Figure S3. Engineering stress-strain curve of PAAm-gel, Se-gel, and Se@PDA-gel.

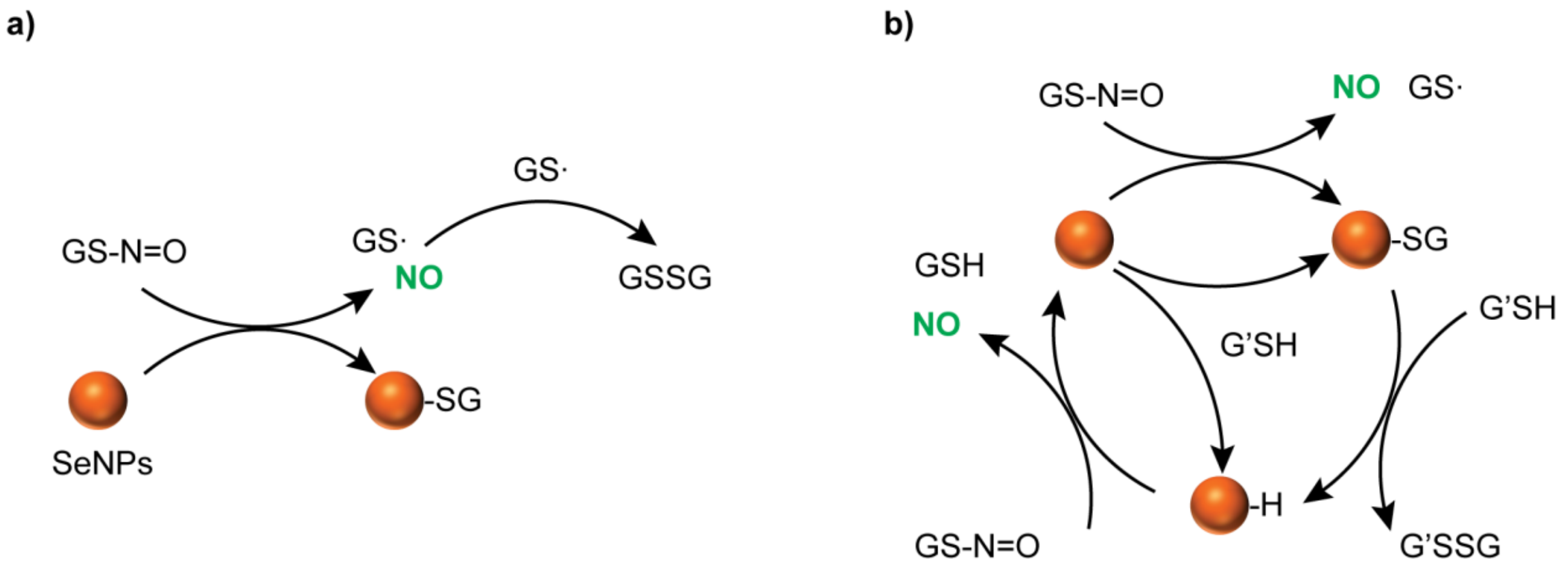

Figure S4. Schematic illustration of SeNP-catalysed NO generation from GSNO in the a) absence and b) presence of GSH.

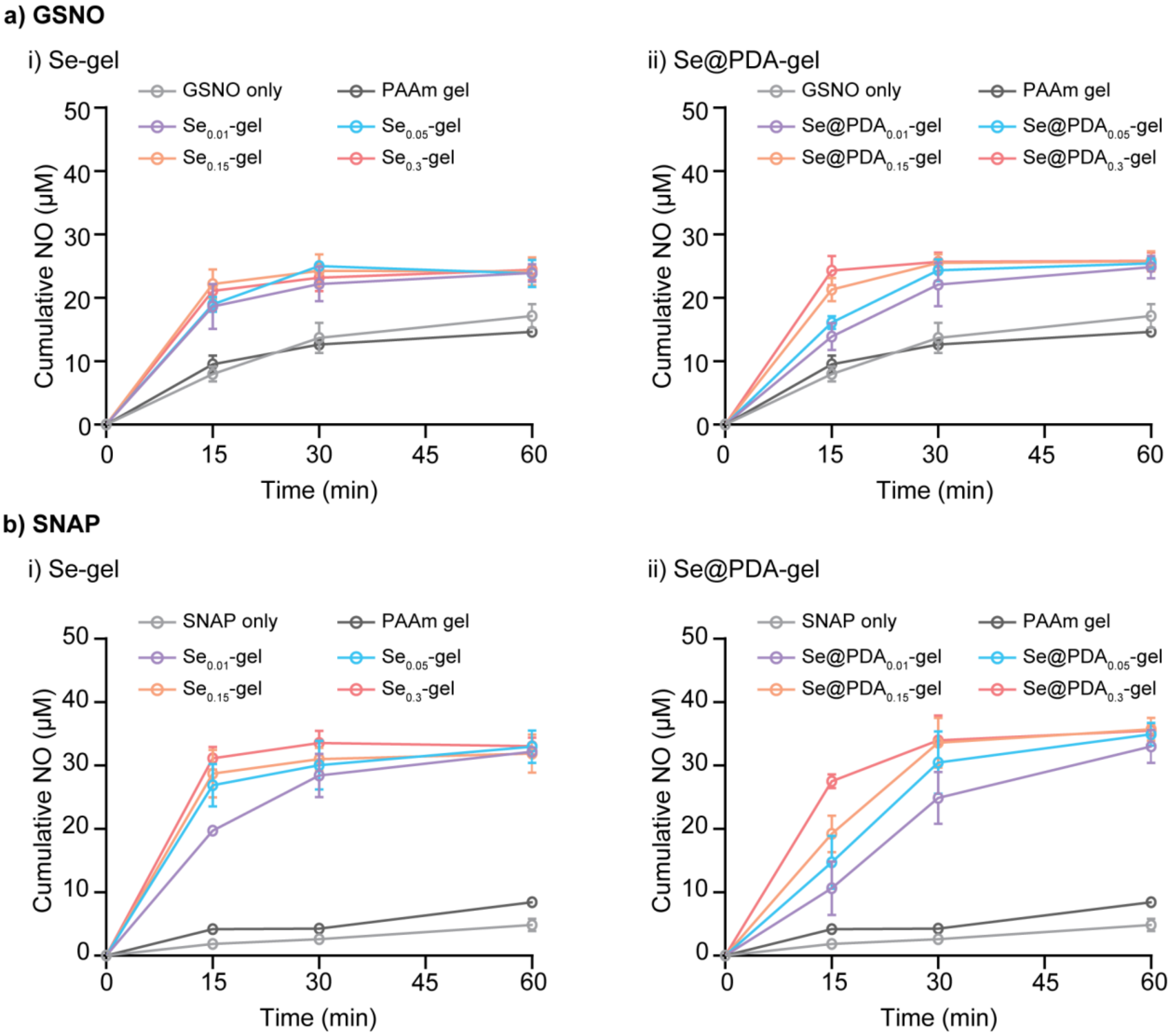

Figure S5. Cumulative NO generation from a) GSNO or b) SNAP (50 μM) catalysed by i) Se-gel or ii) Se@PDA-gel, with NPs loaded at various concentrations (0, 0.01, 0.05, 0.15, or 0.3 mg mL^{-1}) in the presence of GSH (1 mM). n=3; error bars represent standard deviation.

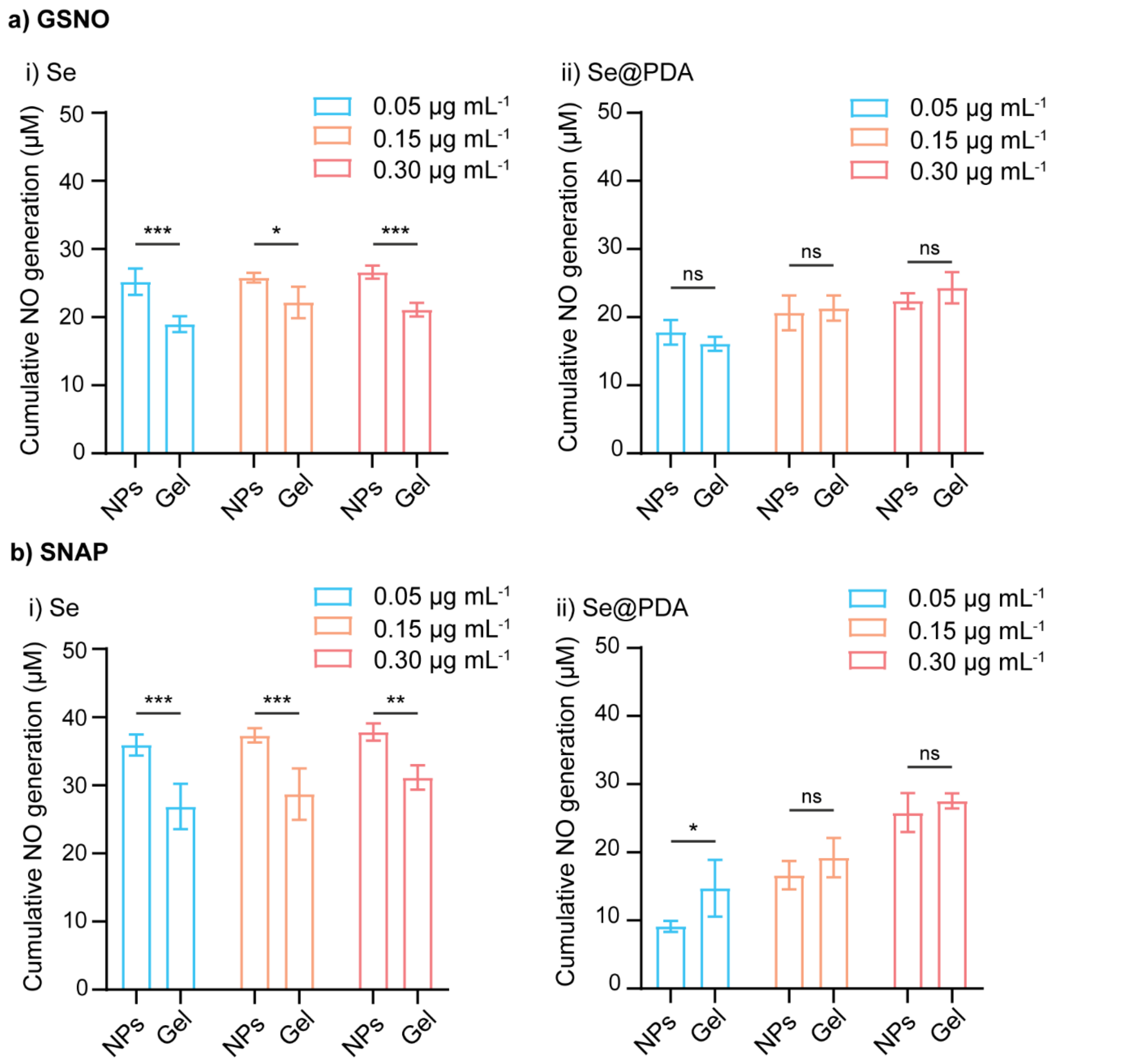

*Figure S6. Comparison of catalytic activity between free NPs and NPs-loaded gel. 15-min cumulative NO generation from a) GSNO or b) SNAP (50 μM) catalysed by i) SeNPs/Se-gel or ii) Se@PDA NPs /Se@PDA-gel with Se content of 0.05, 0.15, and 0.3 μg in 1 mL PBS buffer in the presence of GSH (1 mM). Statistical significance was calculated using two-way ANOVA, ns = no significance, *p < 0.05, **p < 0.01, ***p < 0.001. n=3; error bars represent standard deviation.*

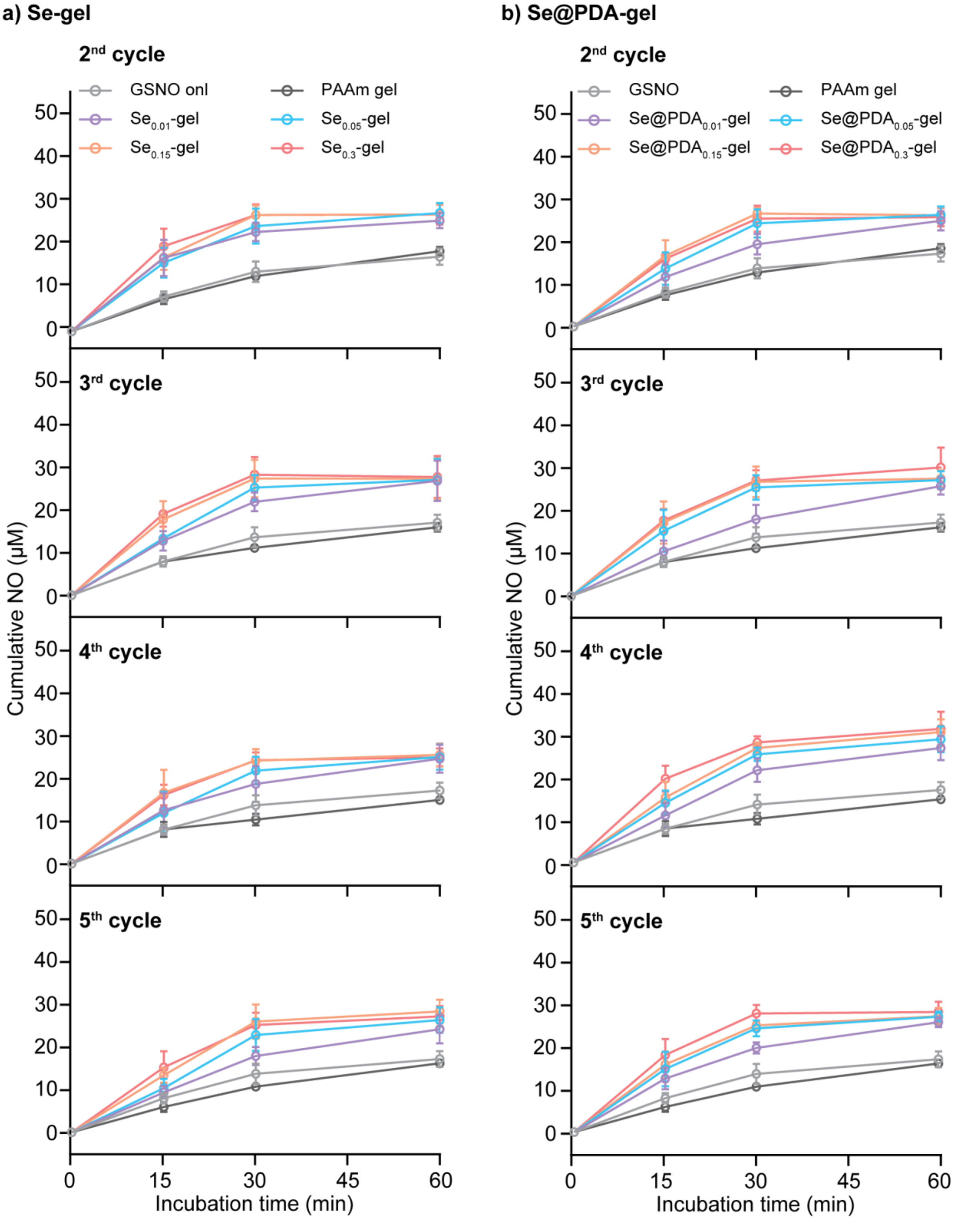

Figure S7. Time-dependent cumulative NO generation from GSNO in the presence of GSH (1 mM), catalysed by a) Se-gel or b) Se@PDA-gel, with various particle loading concentrations (0, 0.01, 0.05, 0.15 or 0.3 mg mL^{-1}) over five reaction cycles. n=3; error bars represent standard deviation.

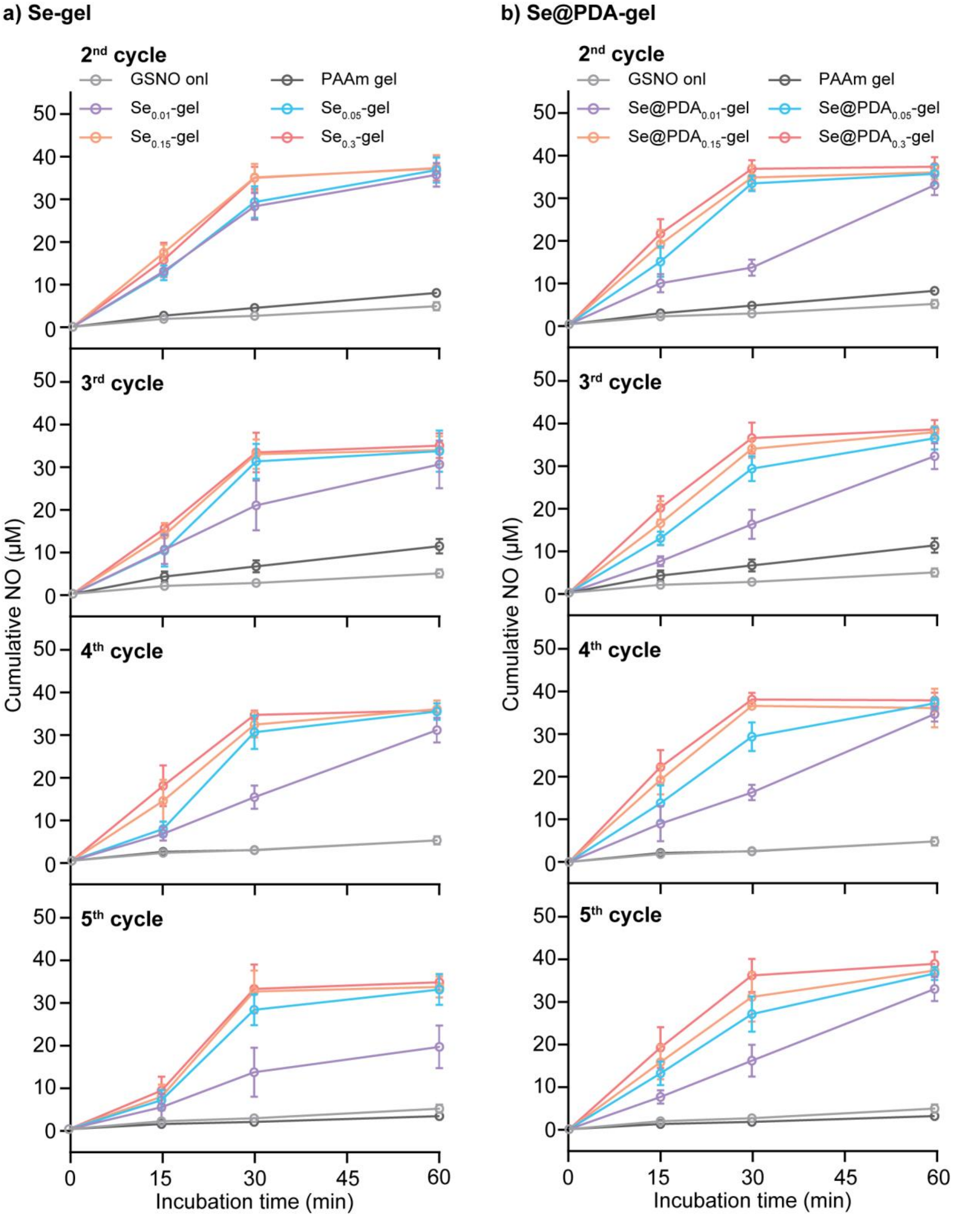

Figure S8. Time-dependent cumulative NO generation from SNAP in the presence of GSH (1 mM), catalysed by a) Se-gel or b) Se@PDA-gel, with various particle loading concentrations (0, 0.01, 0.05, 0.15 or 0.3 mg mL^{-1}) over five reaction cycles. n=3; error bars represent standard deviation.

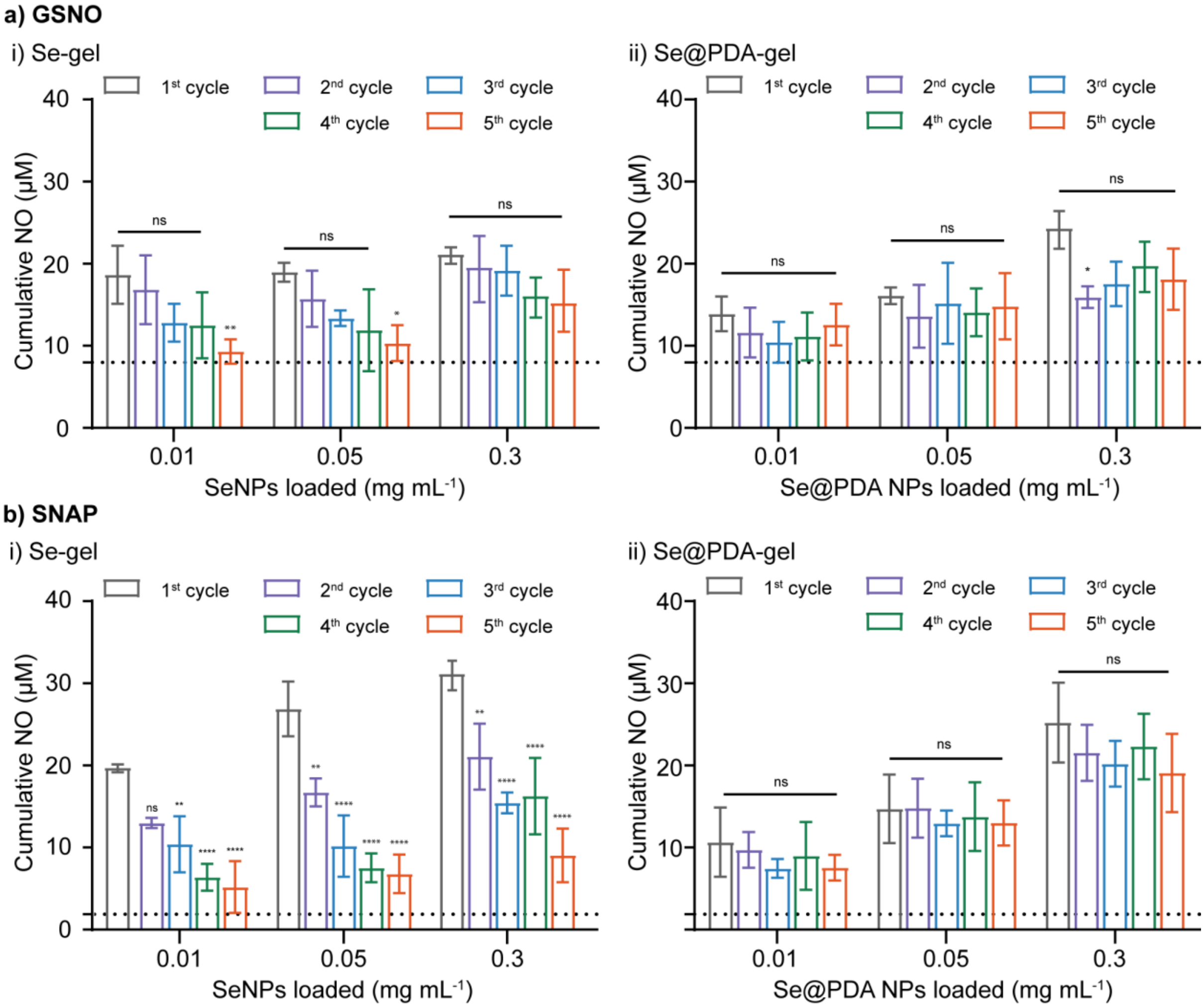

*Figure S9. 15-min cumulative NO generation from a) GSNO or b) SNAP (50 µM) catalysed by i) Se-gels or ii) Se@PDA-gels with NPs loaded at various concentrations (0.01, 0.05, 0.3 mg mL^{-1}) in the presence of GSH (1 mM), over five reaction cycles. The dashed line represents the control (GSNO ot SNAP only) in the corresponding conditions. Statistical significance was calculated using two-way ANOVA, ns = no significance, *$p < 0.05$, **$p < 0.01$, ***$p < 0.001$, ****$p < 0.0001$. n=3; error bars represent standard deviation.*

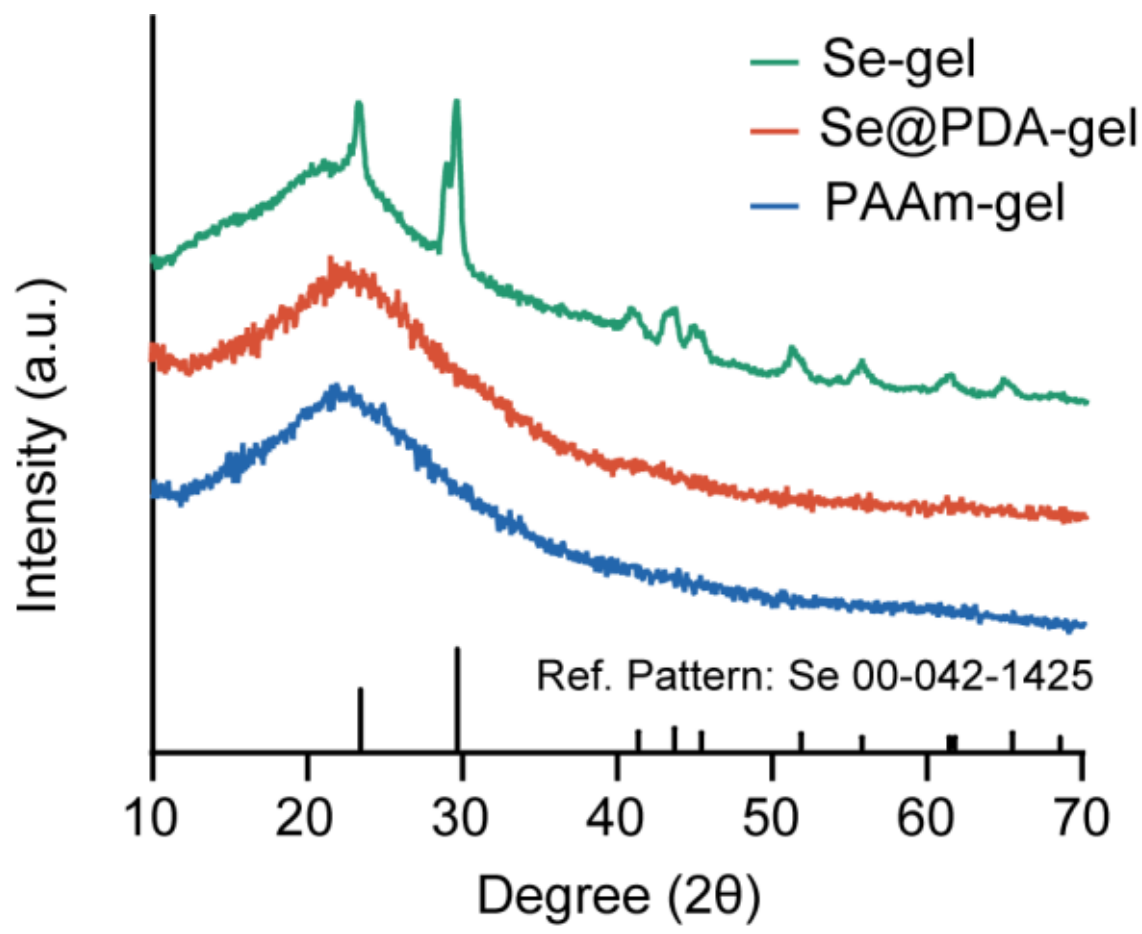

Figure S10. XRD of PAAm-gel, Se-gel and Se@PDA-gel after 24 h incubation in PBS pH 7.4 with GSNO (50 μM) and GSH (1 mM).

Table S4. Se leaching from Se-gel and Se@PDA-gel after various incubation times.

	**Se-gel**		**Se@PDA-gel**	
	**Se [ppb]**	**% Se leaching**	**Se [ppb]**	**% Se leaching**
6 h	22.98	0.0766 %	21.32	0.0711 %
12 h	32.03	0.1068 %	25.41	0.0847 %
24 h	35.89	0.1196 %	35.73	0.1191 %
7 days	44.70	0.1490 %	38.50	0.1280 %

Total mass of Se (10 pieces of 100 μL hydrogels with SeNPs loaded at 0.3 mg/mL):

$$10 \times 100\ \mu\text{L} \times 0.3\ \text{mg/mL} = 0.3\ \text{mg}$$

$$44.7\ \text{ppb} = 0.0447\ \mu\text{g/mL}$$

Mass of Se in 10 mL of supernatant:

$$10\ \text{mL} \times 0.0447\ \mu\text{g/mL} = 0.447\ \mu\text{g}$$

% of Se leaching:

$$0.447\ \mu\text{g}/0.3\ \text{mg} = 0.149\ \%$$

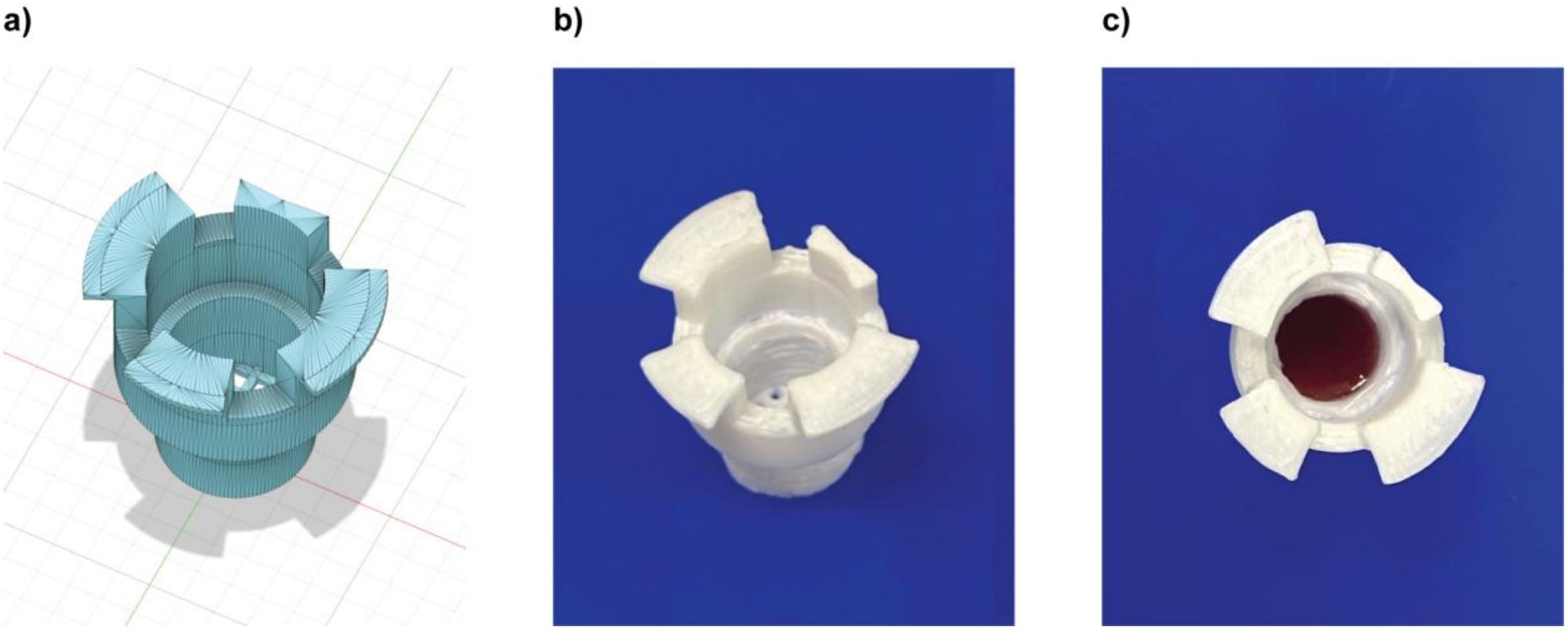

Figure S11. a) 3D model of the hydrogel insert. Optical image of b) the 3D-printed insert and c) the insert with hydrogel placed.

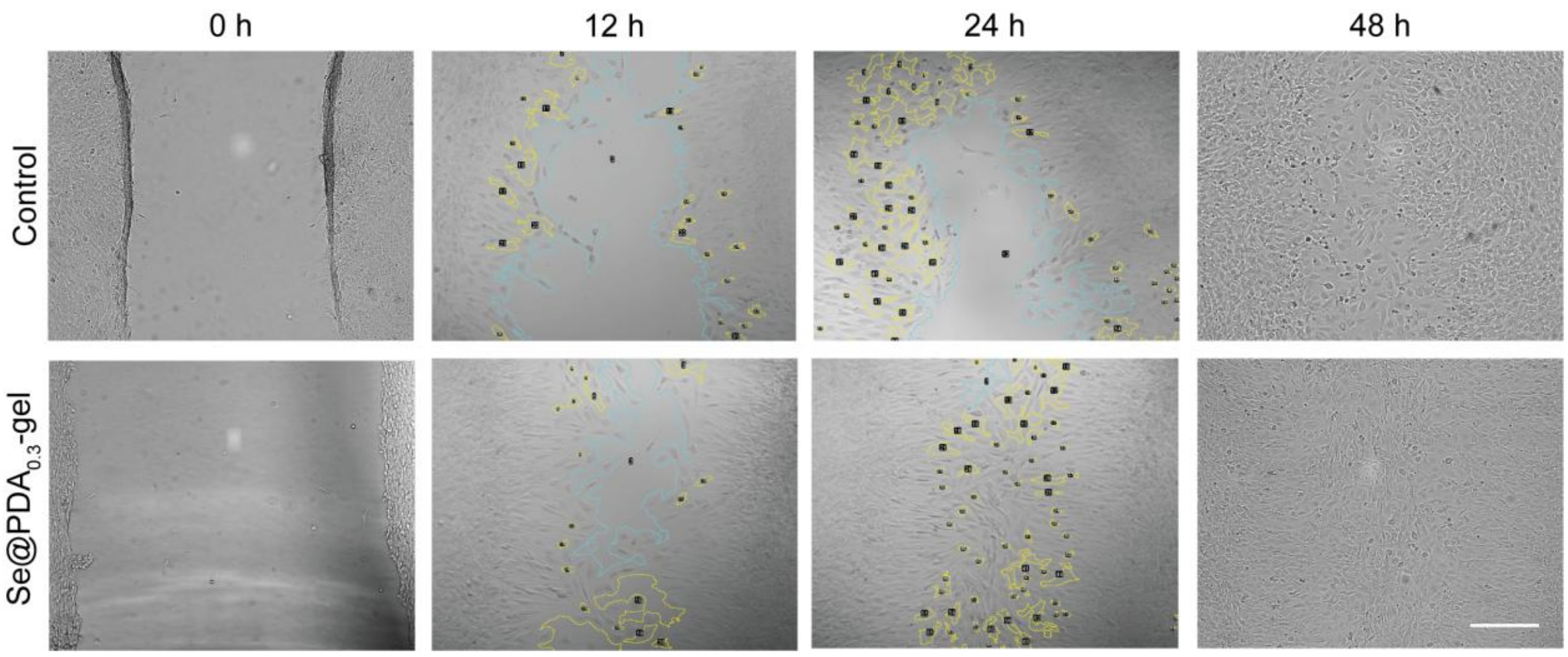

Figure S12. Optical microscopy images of NIH 3T3 fibroblast scratch wound closure in control and Se@PDA$_{0.3}$-gel treated groups over 48 h. Images were acquired at 0, 12, 24, and 48 h after scratch generation (Scale bar 100 µm).

References

1. Sivanantham, M.; Kesavamoorthy, R.; Sairam, T. N.; Sabharwal, K. N.; Raj, B., *Journal of Polymer Science Part B: Polymer Physics* **2008,** *46* (7), 710-720. DOI https://doi.org/10.1002/polb.21402.
2. Gupta, M. K.; Bansil, R., *Journal of Polymer Science: Polymer Physics Edition* **1981,** *19* (2), 353-360. DOI https://doi.org/10.1002/pol.1981.180190214.
3. Samanta, H. S.; Ray, S. K., *Carbohydrate Polymers* **2014,** *99*, 666-678. DOI https://doi.org/10.1016/j.carbpol.2013.09.004.
4. Xu, L.; Che, L.; Zheng, J.; Huang, G.; Wu, X.; Chen, P.; Zhang, L.; Hu, Q., *RSC Advances* **2014,** *4* (63), 33269-33278. DOI https://doi.org/10.1039/C4RA05720A.

5. Cong, H.; Wang, P.; Yu, S., *Small* **2014,** *10* (3), 448-453. DOI https://doi.org/10.1002/smll.201301591.
6. Kaşgöz, H.; Özgümüş, S.; Orbay, M., *Polymer* **2003,** *44* (6), 1785-1793. DOI https://doi.org/10.1016/S0032-3861(03)00033-8.
7. Xiong, C.; Wei, F.; Li, W.; Liu, P.; Wu, Y.; Dai, M.; Chen, J., *ACS Omega* **2018,** *3* (9), 10716-10724. DOI https://doi.org/10.1021/acsomega.8b01205.
